# RAVE-HD: A Novel Sequential Deep Learning Approach for Heart Disease Risk Prediction in e-Healthcare

**DOI:** 10.3390/diagnostics15222866

**Published:** 2025-11-12

**Authors:** Muhammad Jaffar Khan, Basit Raza, Muhammad Faheem

**Affiliations:** 1 Department of Computer Science, COMSATS University Islamabad (CUI), Islamabad 45550, Pakistan; jaffarkulachi@gmail.com; 2 VTT Technical Research Centre of Finland, Maarintie 3, 02150 Espoo, Finland

**Keywords:** artificial intelligence, internet of things, e-Healthcare, heart disease screening and case identification, recursive feature elimination, vanilla recurrent neural network

## Abstract

**Background/Objectives:** Heart disease (HD) is recently becoming the foremost cause of death worldwide, underlining the importance of early and correct diagnosis to improve patient outcomes. Although Internet of Things (IoT)-enabled machine learning approaches have demonstrated encouraging outcomes in screening, existing approaches often face challenges such as imbalanced dataset handling, influential feature selection identification, and the ability to adapt to evolving HD data forms. To tackle the aforementioned challenges, we present a sequential hybrid approach, RAVE-HD (ResNet And Vanilla RNN Ensemble for HD), that combines a number of cutting-edge techniques to enhance screening. **Methods:** Preprocessing phase includes duplicates removal and feature scaling for data consistency. Recursive Feature Elimination is employed to extract the most informative features, while a proximity-weighted random synthetic sampling technique addresses class imbalance to reduce class biases. The proposed RAVE model in RAVE-HD approach sequentially integrates a Residual Network (ResNet) for high-level feature extraction and Vanilla Recurrent Neural Network to capture the non-linearity of the feature relationships present in the HDHI medical dataset. **Results:** Compared to ResNet and Vanilla RNN baselines, the proposed RAVE model attained superior results: 92.06% accuracy and 97.12% ROC-AUC. Stratified 10-fold cross-validation validated the robustness of RAVE, while Sensitivity-to-Prevalence analysis demonstrated stable recall and predictable precision across varying disease prevalence levels. Additional evaluations, including bootstrap and DeLong analyses, showed statistical significance (p<0.001) of the discriminative gains of RAVE. Minimum Clinically Important Difference (MCID) evaluation confirmed clinically meaningful improvements (≥3%) over strong baselines. Cross-dataset validation using the CVD dataset verified robust generalization (92.4% accuracy). SHAP analysis provided interpretability to build clinical trust. **Conclusions:** RAVE-HD shows promise as a reliable, explainable, and scalable solution for large-scale HD screening, consistently performing well across diverse evaluations and datasets. Through statistical validation, the RAVE-HD approach emerges as a practical decision-support tool in HD predictive screening results.

## 1. Introduction

Heart disease is a major worldwide marker of morbidity and mortality, and places a significant burden on e-healthcare systems and individuals  [1]. The complexity of heart disease and a range of risk factors, such as age, lifestyle choices, genetic predisposition and pre-existing conditions, make early diagnosis and effective treatment particularly difficult [2]. Physical examination, laboratory diagnostic assays and imaging studies provide useful information; however, these conventional diagnostic modalities are often time-consuming, costly, and require the expertise of a specialist, and may delay an appropriate intervention. Accordingly, there is an increasing need for efficient and automated machine learning-based tools that can aid in the early detection and screening of HD.

There are some promising advancements in the area of IoT-enabled medical diagnostics with the introduction of artificial intelligence (AI), especially ML [3] and deep learning (DL) [4] methodologies. These methods can work with large, complex data, recognize trends, and provide screening and case identification that can be beneficial to healthcare workers when making intelligent decisions. The fact that AI systems can sort immense volumes of past medical data and learn from it means that the system would be more accurate as well as faster in screening and case identification than the traditional system, as relevant input in clinical decisions [5]. However, there are various limitations associated with employing AI when predicting cardiac disorders.

One of the most substantial problems facing the current medical informatics is the existence of imbalanced datasets [6,7]. Specifically, the prevalence of healthy subjects often overrepresents the prevalence of diseased individuals, leading to a skew that can lead to a bias in screening protocols that can lead to suboptimal performance for the minority class. The literature provides a range of data balancing techniques to address this issue, such as generating synthetic observations and oversampling the minority class. In addition, there are other persistent complexities due to the difficulty in identifying the most informative variables among the myriad of medical descriptors that might affect cardiovascular risk. Redundant [8], unnecessary, or noisy features are a common problem in clinical datasets which can affect the performance of supervised learning algorithms. By focusing on the most salient predictors and using dimensionality reduction techniques, Recursive Feature Elimination (RFE) [9] has identified appreciable increases in predictive accuracy. Furthermore, on the topic of interpretability, the ability to understand the behavior of models is perhaps the single most important quality of artificial intelligence in clinical environments. Physicians need to be able to trust the outputs generated by these systems to incorporate them into routine practice. Models that are opaque “black boxes” without providing any transparency regarding the reasoning behind screening decisions or case identification are often met with skepticism across healthcare environments. In addition to generating precise screening and case identification, AI models also present explanations that are understandable and actionable for healthcare professionals. To rectify the aforementioned issue, we implement Shapley Additive Explanations (SHAP), a method that increases the transparency of the model by figuring out the extent to which each feature plays a role on the model’s screening and case identification  [10].

In the current paper, we propose a highly reliable IoT-enabled HD screening and identification approach that combines multiple advanced methods. We start off by preprocessing the data as rigorously as possible: this includes the balancing of the data with a Proximity-Weighted Synthetic (ProWSyn) sampling-based method to balance the classes. Then, we use the recursive elimination of features algorithm to determine the most relevant features for the optimal selection of features. At the heart of our proposed approach, RAVE-HD (ResNet And Vanilla RNN Ensemble for HD, screening and case identification) lies a hybrid DL architecture with a sequential fusion of Residual Network (ResNet) and Vanilla Recurrent Neural Network (Vanilla RNN). High-level features are obtained by mining HD data using ResNet, while Vanilla RNN is used to learn the non-linear behavior of the features (variables) present in the medically HDHI dataset. With different metrics, we test the effectiveness of our proposed approach and prove that this approach is superior to conventional ML and DL models in terms of screening and case identification quality, which is becoming correct, solid, and understandable.

Contributions:

The key contributions of this article are outlined herein:

- We have proposed RAVE-HD (ResNet And Vanilla RNN Ensemble for HD), a novel sequential deep learning approach, to improve the accuracy of HD screening and case identification. In this approach, a method of Min-Max feature scaling is employed for rescaling the features of the HD dataset so that it makes model stable and improves model’s capability to generalize across different input distributions [11]; ProWSyn (Proximity Weighted Synthetic Sampling) technique is employed to rectify the class imbalance that creates artificial minority-class samples which are closer to majority-class samples, and close to the classification border [12]. Furthermore, this approach also contributes Recursive Feature Elimination (RFE) to extract the most influential features present in the data and to reduce the number of dimensions by using only the most relevant of the features [13]. Next, in this approach, a novel RAVE model that sequentially integrates a Residual Network (ResNet) with high-level feature extraction and Vanilla Recurrent Neural Network (Vanilla RNN) is applied for extracting temporal dynamics from clinical time-series records. Furthermore, by using stratified 10-folds cross-validation, the generalizability of this proposed RAVE model is also improved. Finally, the SHAP is used to improve the trust of the proposed RAVE model. These enhancements improve the generalization capability, reduce bias, and ensure more reliable HD screening and case identification.
- For training objective of the model, a variety of preprocessing techniques are used. These include Min-Max scaling and removing duplicate values. Because these preprocessing procedures reduce noise and use standard data formats, they can help AI algorithms perform better and be more precise. RFE is used to selectively remove irrelevant features, which improves the model’s ability to take advantage of unusual features when predicting HD. Because the model’s generalization skills will be improved, this will reduce the chance of overfitting, reduce the computing cost, and increase the model’s accuracy. The Proximity Weighted Synthetic Sampling (ProWSyn) is introduced to cope with the major problem of the class imbalance. ProWSyn assigns weights to minority samples based on how close they are to the decision boundary. This strategy helps to concentrate more on samples which are more challenging to classify. Therefore, this strategy produces more balanced data that can possibly be utilized to improve the classifier’s screening and case identification of HD without biasing the majority class.
- The sequential hybrid combination of a ResNet and Vanilla Recurrent Neural Network (Vanilla RNN) is proposed in this work. ResNet is used to identify intricate patterns that correlate to the incidence of HD. Vanilla RNN continues it in modeling sequential dependencies, making it useful for time-series and other time-sensitive data and records where the chronology of events’ sequence is crucial. The two models’ sequential hybrid guarantees that the model can use the best aspects of both ResNet feature extraction and the understanding of sequential dependence of Vanilla RNN to provide optimal screening and case identification accuracies. To ensure the strength and generalization of the claimed hybrid model, the stratified 10-Folds cross-validation method (stratified 10-FCV) is implemented. The capability of the claimed model for generalization is more precisely estimated, and the risk of overfitting is minimized. Furthermore, it guarantees constant model performance across various datasets.
- Shapley Additive Explanations (SHAP) is also employed to boost the transparency of proposed RAVE model, by providing information about the influence of each feature over the model’s screening and case identification. SHAP provides a more comprehensive explanation of the model’s choices and provides greater detail, enabling medical experts to trust the model’s results.
- The Sensitivity-to-Prevalence analysis (SPA) is used to address the sensitivity of the RAVE model to the various disease prevalence scenarios. This analytical method ensures that the overall recall rate and precision of the RAVE model remains relatively constant and consistent when applied to clinically diverse screening cohorts, further emphasizing the generalizability and real-world reliability of the model.

The remaining sections of this manuscript are arranged as follows. Section 2 reviews and critically analyzes existing research related to heart-disease screening and case identification with a focus on the application of artificial intelligence (AI)-based methods. Section 3 presents the proposed RAVE-HD approach with the data preprocessing, feature selection, and design of a sequential hybrid deep learning model (RAVE). Section 4 reports the experimental results. Section 4.1 compares the performance of existing machine learning architectures against the proposed RAVE model. Section 4.2 compares feature selection strategies. Section 4.3 analyzes RAVE under different imbalance handling techniques. Section 4.4 is the evaluation of stratified k-fold cross-validation for fair and reliable evaluation. Section 4.5 is about statistical analyses for significance. Section 4.6 is about sensitivity to prevalence for robustness evaluation on original data. Section 4.7 is about the generalizability gap: cross dataset evaluation for heart disease screening. Section 4.8 is about SHAP interpretability for HD prediction and case identification. Section 4.9 is about ablation study for the contribution of each component. Section 5 discusses limitations and also describes future work. Finally, Section 6 wraps up the RAVE-HD approach study.

## 2. Related Work

The most recent research on HD prediction, screening, and case identification has been explored with data balancing, feature selection, ML, and DL techniques to achieve higher accuracy and to limit the existing problems, such as dealing with data imbalance, feature selection, and the identification of intricate patterns.

Various ML and DL architectures have been reviewed to improve the prediction of heart diseases, especially when it comes to class imbalance in the medical records. As stated in the research findings of Bilal et al. (2025) in [14], a hybrid DL model was developed that incorporates Bidirectional Long Short-Term Memory (BiLSTM) network with Bidirectional Gated Recurrent Units (BiGRUs), and demonstrated improved performance compared to conventional models. In order to tackle the matter of dataset imbalance, the Synthetic Minority Oversampling Technique (SMOTE) was implemented to the dataset by creating artificial samples of minority classes, hence, diagnosing with better accuracy and decreasing the likelihood of overfitting. However, this dependency on SMOTE can be rather problematic to deal with when it comes to extremely imbalanced classes or extremely small datasets, with a risk of causing diminished generalization ability.

The prediction of HD using ML has also been used more and more in the academic literature, especially with specific references to data imbalance and feature selection. The PaRSEL (a concatenation stacking (P) of Passive Aggressive Classifier (PAC), Ridge Classifier (RC), Stochastic Gradient Descent Classifier (SGDC), and eXtreme Gradient Boosting (XGB)) with a Logit Boosting (LB) meta layer proposed by Noor et al. (2023) is a stacking approach to this screening and case identification task [15]. The aim of class imbalance mitigation affords the researchers a combination of three data-balancing algorithms: Proximity Weighted Random Affine Shadowsampling (ProWRAS), Localized Random Affine Shadowsampling (LoRAS), and SMOTE, and parallelizing them with strategies of dimensionality reduction such as Recursive Feature Elimination (RFE), Linear Discriminant Analysis (LDA), and Factor Analysis (FA). By their experimental results, they provide evidence that the model based on the PaRSEL model is more accurate than the classification of its inner components, reaching up to 97% accuracy and 98% Receiver Operating Characteristic Area under the Curve (ROC-AUC), hence proving the efficacy of merging a variety of balancing and dimension-reduction methods. However, the authors face several limitations, including the potential for overfitting the model when dealing with higher-dimensional data and the considerable processing overhead required to execute it.

Some techniques of data balancing have been developed to solve the problem of class imbalance in the prediction of HD. The new divide-and-conquer approach towards data balancing proposed by Yang et al. (2024) utilizes the *K*-Means algorithm [16]. This algorithm splits the data into several clusters, and each group is sampled separately to balance the classes. Compared to the traditional methods, the authors compare this approach to SMOTE, NearMiss, and SMOTE Tomeklinks (SMOTE-Tomek). This proposed technique had a great impact on classifier performance, as it only increased the accuracy to 81 to 90 percent. However, the method’s limitation is that it uses clustering, which may cause issues with substantially overlapping instances of classes within the classes. In the case of complex medical datasets, such conditions can lead to overfitting of the predictive algorithm and introduce bias in the generation of synthetic data.

Research on coronary heart disease has been gaining momentum in recent years and ML algorithms are used for prediction. In the study by Omotehinwa et al. (2024) [17], the authors aimed to optimize the Light Gradient Boosting Machine (LightGBM) algorithm for the early detection of CHD. Their approach included known class imbalance mitigation by Borderline-SMOTE and missing data imputation by Multiple Imputation by Chained Equations (MICE). The resulting, optimally tuned model gave an accuracy rate of 98.82 per cent, and a Receiver Operating Characteristic Area under the Curve (ROC-AUC) of 0.9963, and a precision of 98.35%. This is a significant improvement over baseline models. The downside of the model is, though, that it requires computationally costly hyperparameter tuning, and it can be overfitted when using synthetic samples, which can result in impaired generalization to other datasets.

Manikandan et al. (2024) proposed a machine learning-based prediction of heart disease using Boruta feature selection algorithm and classification methods like Logistic Regression (LR), Support Vector Machines (SVMs) and Decision Trees (DTs) [18]. In their study, their analysis used Boruta to select the best features of HD dataset acquired in the Cleveland Clinic and showed that it enhanced the model functioning of the dataset to a high level of accuracy of 88.52 percent with LR. However, LR has a limitation, as it assumes linear relations, and hence it might fail to produce an ideal pattern of the data due to complex non-linear relations, especially lessening its performance compared to more complicated models.

Various ML models such as RF, LR, Gaussian Naive Bayes (GNB), and LDA were covered by Malakouti et al. (2023) in the classification of HD samples based on Electrocardiogram (ECG) data [19]. It proved that the GNB classifier presents the best performance of classification accuracy of 96% of the healthy population and 96% of the HD population. However, a limitation of GNB is that It makes a hypothesis that features are independent, and a normal distribution is followed by continuous features, which is not always the case when dealing with complex ECG data and may sometimes reduce accuracy.

Ali et al. (2019) suggested a hybrid diagnosis system of feature selection relying on the $\chi^{2}$ statistical model fused with a Deep Neural Network (DNN) optimally configured to predict HD [20]. The $\chi^{2}$-DNN model had a prediction accuracy gain of 93.33%, compared to traditional DNN models since the model removed the irrelevant feature and had no overfitting. The drawback is that a DNN model might overfit if it is employed improperly with too many layers or noisy features, but this can be somewhat mitigated by the rigorous Grid Search Optimization (GS).

Yongcharoenchaiyasit et al. (2023) suggested a Gradient Boosting (GB)-based model for classifying elderly patients with dementia aortic stenosis (DOS) and heart failure (HF) [21]. Other classifiers showed that the model performed with a major improvement, with accuracy of 83.81%, with the use of feature engineering. Even though GB produces outstanding results, its condition with respect to class imbalance is its main limitation: unless particular procedures like oversampling or changing the class weights are added, it is more likely to provide biased predictions.

Feature selection strategies were employed by Noroozi et al. (2023) to optimize ML models to predict HD [22]. This experiment confirmed that feature selection enhanced the accuracy, precision, and sensitivity of ML models that include SVM, GNB, and RF. Nevertheless, GB was proved to be restricted in its sensitivity towards overfitting, as GB used with some form of feature selection would perform poorly, particularly in cases where the number of features was too big or contained irrelevant features. This obstacle impaired the capacity of the model to generalize within dissimilar datasets adequately.

Ben-Assuli et al. (2023) came up with a human–machine collaboration framework to model prediction models that can lead to accurate Congestive Heart Failure (CHF) risk stratification [23]. Their experiment compared feature sets as chosen by the experts and the ML models, with the combined list of expert and ML-chosen features performing better than either of them alone, with an ROC-AUC of 0.8289. Although these have positive developments, one of the limitations of the method is that the feature set used is complex, with the final model having 42 features that might cause an overload of information on clinicians in practice. Moreover, the model is based on the adherence to such a large amount of data, which brings about the issue of computational efficiency and scalability in smaller institutions.

Pavithra et al. suggested a hybrid method of feature selection, HRFLC (RF + Adaptive Boosting (AdaBoost) + Pearson Coefficient), to forecast cardiovascular diseases [24]. It is the combination of the merits of several algorithms with the purpose of enhanced prediction accuracy, using the finest features of a large amount of information. The results of the HRFLC model had a higher rate of accuracy and less overfitting than the conventional ones. However, one of the drawbacks of this strategy is its computational complexity, particularly when employing ensemble techniques like RF and AdaBoost, which may strain the resources as well as wasting time when dealing with large-scale data.

A multi-modal strategy for heart failure (HF) risk estimation was suggested by Gonzalez et al. (2024) [25], utilizing brief 30 s ECG findings merged with sampled lengthy-duration Heart Rate Variability (HRV). The models they used to achieve survival are survivors like XGB and Accelerated Failure Time (AFT) with a Residual Network (ResNet) model, which captures raw ECG signals directly. This study generated a better result in the HF assessment of risk with a 0.8537 concordance index. However, the ResNet model’s complexity is one of its limitations; it requires a lot of processing power and may not even be as interpretable as other models, making it more difficult to apply to devices with constrained resources.

Al Reshan et al. (2023) presented an effective HD prediction model comprised of Hybrid Deep Neural Networks (HDNNs), which blend dense-layered LSTM networks with CNN to enable the model to achieve a greater degree of accuracy [26]. Their model attained 98.86% accuracy on complete datasets and performed well in contrast to traditional ML models. However, the computational cost of the HDNN technique is one of its limitations, which makes it inappropriate for use in real-time or resource-constrained environments, especially when managing a big database is required as is to be anticipated as the volume of the medical dataset increases.

The challenge of HD prediction was approached hybridically by Shrivastava et al. (2023) using a CNN and BiLSTM network combination to boost classification performance [27]. The model’s recorded accuracy on the Cleveland Heart Disease (CHD) dataset was outstanding at 96.66%. One drawback of this strategy is its computational cost, particularly the deep design of the hybrid model, which makes it unsuitable for real-time implementations on devices with constrained processing power.

Although there have been advancements in the prediction, screening, and case identification models of HDs, there has been a serious demand for better methods concerning data balancing, feature selection, and hybrid ensemble approaches. Balanced data make sure that minority classes are represented enough, and the model does not provide improper bias to the majority class. Decent feature selection strategies must be employed to downscale the dimensions of the information, yet perform the best in extricating the most applicable information for the screening and case identification. Furthermore, the hybrid approach of an ensemble of multiple models has the capability of improving the robustness and generalizability of the screening and case identification system. Besides these, it is important to introduce Explainable AI (XAI) methods to make the work of complex models more transparent and understandable. XAI also allows healthcare practitioners to comprehend and believe in decisions made by the model, an essential aspect of clinical adoption. From the above literature review, we advocate for future enhancements in the capability of heart disease screening and case identification models. These models should incorporate advanced computational techniques to improve diagnostic accuracy, enhance generalization, and make heart disease screening or case identification more reliable and effective.

## 3. Proposed System Approach: RAVE-HD (ResNet and Vanilla RNN Ensemble for HD) Screening and Case Identification

**Figure 1:**
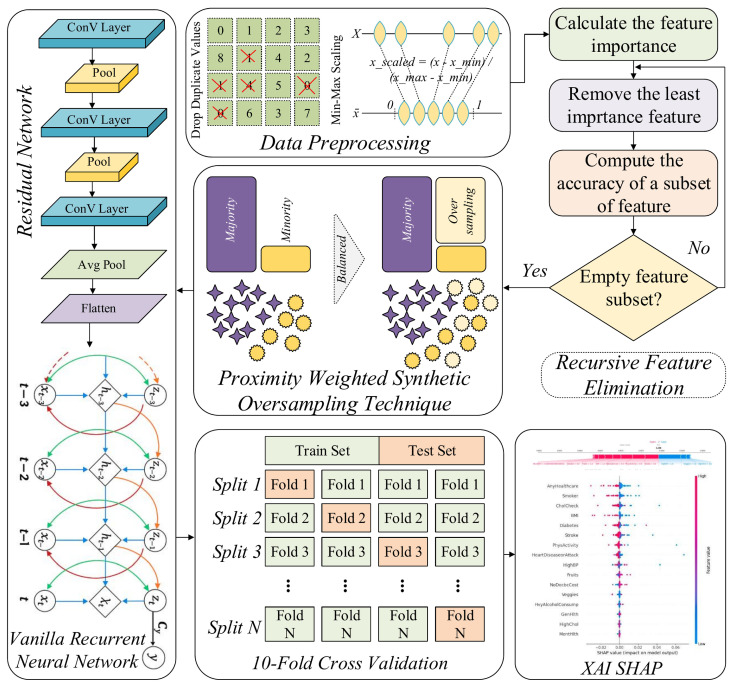
RAVE -HD (ResNet and Vanilla RNN Ensemble for HD screening and case identification), proposed system approach for heart disease screening and case identification.

In this work, we present a RAVE-HD (ResNet And Vanilla RNN Ensemble for HD screening and case identification), a novel sequential DL approach for screening and case identification of HD that integrates multiple data preprocessing and modeling stages. Initially, duplicates records are identified and removed for data redundancy avoidance. Subsequently, Min-Max scaling is employed to normalize all numerical features within a unified range, facilitating stable and efficient model convergence. To mitigate the issue of class imbalance inherent in the dataset, the Proximity Weighted Synthetic Oversampling Technique (ProWSyn) is utilized, which generates synthetic minority class samples near the decision boundary, consequently improving the ability of the approach to identify rare heart disease cases. Following balancing, we adopt Recursive Feature Elimination (RFE) to reduce dimensionality by selecting the most influential features, which helps in minimizing noise and overfitting. Finally, we propose a hybrid DL model that sequentially combines the powerful spatial abstraction capabilities of the Residual Network (ResNet) with the temporal sequence modeling strength of the Vanilla Recurrent Neural Network (Vanilla RNN) to capture complex feature interactions and temporal dependencies as shown in Figure 1, and illustrated by Algorithm 1.

### 3.1. Data Preprocessing

In order to make the HDHI dataset suitable for the effective training of deep learning models, an elaborate structured preprocessing pipeline is implemented to increase the quality, consistency, and intelligibility of the dataset. These steps ensure that all the features are in the correct formats and in similar scales, which will ensure stability and efficiency in the further model training process.

### 3.2. Data Description

This study uses a vital dataset called the Heart Disease Health Indicators (HDHI), which is a rich dataset developed for cardiovascular health analytics and predictive modeling of cardiac disease. The HDHI is derived from the Behavioral Risk Factor Surveillance System (BRFSS) [28], a longitudinal surveillance program that was begun in 1948 and is currently managed by the U.S. Centers for Disease Control and Prevention (CDC). The dataset is made up of 253,680 unique records, each containing the health profile for a unique adult respondent. It includes 22 variables ranging from demographic characteristics, lifestyle behaviors, and clinical health measures, with notable characteristics being Age, Body Mass Index (BMI), Smoking behavior, Alcohol use, and Physical and Mental health status, as well as the history of Stroke and Diabetes. The data were collected through standardized, self-reported questionnaires and health surveys conducted under the CDC’s BRFSS initiative, ensuring consistency and comparability between all features of the participants.

All respondents, aged 18 years or older with complete and valid responses to the 22 health indicators, were included in the study with respect to the inclusion criteria. To maintain the integrity of the dataset and to avoid the introduction of bias in model training, records containing missing data, duplicate records or values above or below the range were removed in line with documented exclusion criteria.

The operational definition of “heart disease” followed the guidelines from CDC; participants who reported a diagnosis of coronary heart disease, myocardial infarction, or angina were assigned a code of 1 (positive case of heart disease), while all others were assigned a code of 0 (negative case of heart disease). Given that the data is tabular, it is very suitable for binary classification problems. Its great width, demographic heterogeneity, and open availability via the Kaggle platform make it a reliable reference for public health analytics and modeling using machine learning.

#### 3.2.1. Duplicate Entries Identification and Removal

Since the HDHI dataset consists of real-life records, it is important to identify the redundancy of the data since such redundancies can distort the data and mislead the learning process. A full analysis of all twenty-two columns revealed 23,899 duplicate records in the dataset. All these redundant entries were carefully removed resulting in a curated dataset of 229,781 unique observations. The maintenance of the consistency and representativeness of the patient population was ensured by retaining a single example of each record. This step of data cleaning is essential for models to be able to identify real patterns in the data. By removing repetitions, the models avoid memorizing duplicate information. As a result, they can generalize better and perform more reliably on unseen cases.

#### 3.2.2. Min-Max Features Scaling

Range normalization (Min-Max Features Scaling or Rescaling or Normalization) is the most straightforward method for rescaling any numerical feature into a specific range, usually [0, 1], which is achieved by the normalization of the features. It is a normalization preprocessing step that is especially necessary when there is a dataset consisting of features measured on different units or scales. Since such inconsistencies might have a disproportionately significant impact on the model training, this step is of vital importance, notably in algorithms that follow a gradient descent or those based on Euclidean distance. Through the standardization of the range of each feature, Min-Max scaling makes sure that a single feature cannot dominate the learning process if the shift happens only due to the scale, which in turn stabilizes the optimization path and accelerates convergence during training. The transformation is performed by subtracting a feature’s minimum (lowest) observed value and dividing by the difference between both of the maximum and minimum numbers as explained by the following Equation (1):

(1) $$x^{\prime }=\frac{x-x_{\min}}{x_{\max}-x_{\min}}$$

In the equation, *x* represents the value of the original feature, $x_{\max}$ and $x_{\min}$ are parts of the feature that have the maximum and minimum threshold numbers, respectively, and $x^{\prime }$ is the normalized output. This linear scaling procedure guarantees that all of the feature values are in the same number range, so they can make equal and proportional contributions to the model’s training process. Therefore, Min-Max normalization makes the model more numerically stable and boosts the capability of the model to generalize across different input distributions [11].

### 3.3. Clinical and Machine Learning-Adapted Baselines

Traditional cardiovascular risk scores, such as the Framingham Risk Score (FRS), Atherosclerotic Cardiovascular Disease (ASCVD), and SCORE2, estimate long-term CVD risk using laboratory and physiological parameters, including systolic blood pressure, HbA1c, creatinine, and blood lipid levels (LDL-C, HDL-C). These clinical variables are not available in the HDHI dataset; therefore, a direct numerical comparison with these established calculators is not feasible. SCORE2 (SCORE2 (Systematic Coronary Risk Evaluation 2) is a European 10-year cardiovascular risk model based on age, sex, blood pressure, cholesterol, and smoking status; key inputs such as systolic BP and cholesterol are missing in the HDHI dataset) is also excluded for this reason.

To provide a clinically meaningful reference, the proposed RAVE-HD approach’s suggested RAVE model is benchmarked against a set of machine learning-adapted models that emulate conventional risk logic using survey-based features. These include Logistic Regression (LR), Naïve Bayes (NB), Deep Belief Network (DBN), Gradient Boosting (GB), Residual Network (ResNet), Vanilla RNN, and the ensemble EnsCVDD. These models are widely applied in cardiovascular screening and risk stratification using health survey data.

Traditional risk equations use fixed mathematical terms to represent interactions between variables (e.g., age × cholesterol). RAVE-HD does not require such predefined terms; instead, it learns these complex dependencies directly from the data through its hybrid ResNet-VRNN architecture and explainable feature selection layer. This design enables the unbiased discovery of behavioral–clinical relationships without relying on manually specified clinical coefficients. The comparative evaluation of RAVE-HD against these clinical and machine learning baselines is presented in “Effect Size and Clinical Relevance: MCID *(Minimum Clinically Important Difference) Analysis” Section*, highlighting its predictive performance, interpretability, and clinical relevance.

### 3.4. Data Balancing

In the case of classification tasks that involve medical data, one of the most significant and still not fully solved issues is that the classes are imbalanced. This problem was detected as the root of the reliability and fairness problems of machine learning models. This challenge gains more significance in DL settings, where the optimization process may inherently favor the majority class due to the disproportionate distribution of class labels. When predicting heart disease, the dataset that this study is based on has a notable imbalance, as very few cases of heart disease, only about (9%), are labeled out of the whole dataset, while the rest, (91%), are labeled as healthy. The distributions that are skewed like that frequently give rise to models that are not good at recognizing minority class examples and hence are likely to miss serious medical conditions, although they may seem to perform well according to overall accuracy.

To reduce this drawback, we implement the ProWSyn as a directed method for equalizing the training data. ProWSyn is a more sophisticated and efficient option than classical methods such as SMOTE. Its goal is to make artificial samples of minority class in a more intelligent and context-aware manner [12]. Whereas SMOTE generates new instances via the linear interpolation among randomly chosen minority neighbors, ProWSyn applies the proximity-aware weighting system that continuously gives the most number of points to those minority examples which are generated closely to the boundary of making a decision, where the classification is usually most uncertain. This target sampling method thus guarantees that the created synthetic examples are not only varied but also informative; they basically strengthen the decision boundary and provide the model with greater sensitivity to minority class examples that are challenging to classify. Consequently, in addition to enhancing the model’s overall screening and case identification robustness, ProWSyn guarantees its sensitivity and fairness, which are the most important conditions in domains with high stakes like medical diagnostics.

Rationale for Selecting ProWSyn: The preference for ProWSyn methodology over conventional oversampling techniques like SMOTE and ADASYN is based on the fact that it combines the use of proximity-based weighting with the dynamic boundary awareness. In contrast to SMOTE, which generates interpolations between minority instances uniformly, or ADASYN which generates interpolations based on density distributions, ProWSyn deliberately pays attention to minority observations which are near to the decision boundary by giving them greater proximity weights. As a result, the ability of the model to synthesize more informative artificial instances improves its discriminative ability in boundary-rich regions. Moreover, ProWSyn has the following features: an exponential decay of weights to reduce the weight of more remote samples, and controllable partitioning parameters (L, K) to allow domain-specific fine-tuning. As a result, ProWSyn prevents the over-concentration of synthetic points, limits redundancy, and achieves a process of sampling that is balanced and sensitive to nuances at boundaries, which makes it particularly suitable for medical datasets where decision boundaries are often subtle and clinically important.

The central idea of ProWSyn is to assign a higher probability of selection to those minority class instances that are closer to majority class samples. This proximity is quantified using a distance-based weighting function. For each minority class instance $x_{i}$, the proximity weight $w_{i}$, using Equation (2) is calculated as the inverse of its Euclidean distance to the nearest majority class instance $x_{j}$:

(2) $$w_{i}=\frac{1}{1+d(x_{i},x_{j})}$$

These weights have to be normalized across all N minority instances in order to obtain probabilities for sampling by using Equation (3):

(3) $$P\left(x_{i}\right)=\frac{w_{i}}{\sum_{k=1}^{N}w_{k}}$$

After the probabilities are determined, a random factor λ∈[0,1] is introduced, and the interpolation method is used between a selected minority instance $x_{i}$ and one of its neighbor instances $x_{n}$ belonging to the same minority class to generate new synthetic instances as calculated by using Equation (4):

(4) $$x_{\mathrm{syn}}=x_{i}+\lambda \cdot \left(x_{n}-x_{i}\right)$$

The interpolation strategy guarantees that synthetic instances are created in areas of higher classification uncertainty and so strengthen the decision boundary between classes. By amplifying samples which are hard to learn, ProWSyn helps to increase the minority class sensitivity while maintaining the overall classification robustness. As a result of applying ProWSyn, the class distribution becomes more balanced, reducing the influence of the majority class and allowing the DL model to train for representative features for both healthy and heart disease cases.

### 3.5. Recursive Feature Elimination

Feature selection is a principal factor to raise the performance and understandability of a DL model, primarily when dealing with high-dimensional healthcare datasets. This process removes unnecessary or redundant features, reducing the number of features that are used, thus reducing the model complexity, preventing overfitting, speeding up the training, and, most importantly, improving the capability of the model to make it more generalizable to newly acquired data. In this study, we use RFE [13] as a principal and systematic method to optimize the input feature space before feeding it into the proposed hybrid DL model.

RFE is a backward selection algorithm, which starts with all the input features and goes on to remove those that are judged to be of least importance for the model’s capability for making predictions, screening, and case identification. To decide the relevance of each and every feature to the problem (e.g., based on classification loss or model weights), a new model is trained on the given subset and the importance of each feature is estimated. The feature that has the contrary effect is eliminated, and the model is trained once more using the rest of the samples subset. This operation is used again and again, until a reliable number of the most key important features are left. By consistently identifying and retaining the most informative attributes, RFE ensures that the final selected subset maximally supports the learning objective while reducing noise and dimensionality in the data. Mathematically, let the initial feature set be represented as $F=\{f_{1},f_{2},\ldots ,f_{21}\}$. For each feature $f_{i}$, an importance score $I(f_{i})$ is calculated using (5), by measuring the change in a performance criterion (e.g., classification loss L) when the feature is removed:

(5) $$I\left(f_{i}\right)=L\left(F\setminus \left\{f_{i}\right\}\right)-L\left(F\right)$$

Every iteration removes the feature with the lowest significance score by using Equation (6):

(6) $$F^{\left(k+1\right)}=F^{\left(k\right)}\setminus \left\{\arg\min_{f_{i}\in F^{\left(k\right)}}I\left(f_{i}\right)\right\}$$

This recursive procedure continues until an optimal number of features, denoted with $|F^{*}|=15$, is retained. The resulting optimal subset $F^{*}$ includes the most influential features supporting the model’s capability to distinguish regarding cardiac disease and healthy (normal) examples. Through RFE, the initial 21 features are reduced to 15 key predictors: HighBP, CholCheck, BMI, Smoker, Stroke, Diabetes, PhysActivity, Fruits, NoDocbcCost, GenHlth, MentHlth, PhysHlth, Sex, Age, and Education. These features demonstrate consistent relevance across multiple iterations and directly enhance the capacity of the proposed hybrid DL model to determine intricate non-linear associations present in the data.

### 3.6. Residual Networks

ResNets are a subclass of Deep Neural Networks (DNNs) that address the degradation problem often encountered in very deep architectures. The accuracy of training a traditional neural network also saturates as the number of layers grows and subsequently falls off dramatically because of the vanishing gradient problem and the inability to optimize the deeper layers [29]. ResNet addresses this problem by incorporating shortcut connections that enable residual mappings to be learned by such a network as opposed to attempting to learn the underlying desired mapping, directly.

The residual block, which enables the input to be fed straight to the output without passing through one or more layers, is the core component of ResNet. The ResNet instructs the network to capture a residual function F(x)=H(x)−x mathematically, rather than a direct mapping H(x). This leads to Equation (7).

(7) H(x)=F(x)+x

Here, *x* is residual block’s input, F(x) is the residual mapping that was discovered by a sequence of activation, convolutional, and batch normalization layers, and H(x) is the final value of the output. By allowing gradients to pass straight through the skip connections during backpropagation, this additive identity mapping makes deep network training more reliable and effective. A common residual block has two or three convolutional layers, preceded by (and a non-linear activation function like ReLU followed by) batch normalization. When the input and the output sizes of a residual block do not match, a linear projection is used to fix dimensions in the shortcut pathway. This is normally performed by a 1 × 1 convolution that introduces a learnable transformation in order to be dimensionally equalized. The redesigned residual mapping may be written as in the form of Equation (8):

(8) $$H\left(x\right)=F\left(x\right)+W_{x}$$

where F(x) is the output of the residual function, and $W_{x}$ is a trainable weight matrix and serves as the input *x* bypassing the connection. The adjustment makes residual connection additive and still has the flexibility to architecture.

This kind of design permits the ResNet architecture to be well scaled to be very deep, where very deep networks like ResNet-50, ResNet-101, and ResNet-152 can be built without the performance penalty of vanishing gradients. Training in even very deep networks can be stabilized by the use of projection or identity skip connections, allowing efficient overall training. Also, the residual learning technique gives the network the capability to learn low-level and high-level abstractions at each layer, and thus ResNet is extremely useful in various applications such as image classification, time-series prediction, etc. With such advantages, ResNet has become a building block of DL models, with exceptional accuracy performance, robust convergence, and strong generalization properties.

### 3.7. Vanilla Recurrent Neural Networks

Vanilla RNNs belong to an important group of neural networks whose specific goal is the representation of sequential information, through learning the architecture of temporal dependencies between the elements of a sequence. Unlike the feedforward neural networks, where each of the inputs is processed separately with no attention to the preceding context, Vanilla RNNs have recurrent connections that allow the neural networks to keep the memory records of the past time steps. This recurring process enables the model to model the time dynamics and the context-based relationship in a sequence and as such, this model can be effectively applied in tasks that have time-dependent data. Consequently, Vanilla RNNs have found extensive use in applications in the domains of physiological signal analysis, and sensor-based time-series modeling where the relative order and development of input features is of central importance in making an effective prediction.

The Vanilla RNN processes an input sequence $\left\{x_{1},x_{2},\ldots ,x_{T}\right\}$ over *T* time steps, where each $x_{t}\in R^{n}$ is vector of an input at time *t* [30]. The main calculations are recursively updating the hidden state $h_{t}\in R^{m}$ with both the input at present time step and the input from the previous time step:

(9) $$h_{t}=\phi \left(W_{xh}x_{t}+W_{hh}h_{t-1}+b_{h}\right)$$

(10) $$y_{t}=W_{hy}h_{t}+b_{y}$$

In this case, $W_{xh}\in R^{m\times n}$ is the weight matrix (WM) of the input-to-hidden (ITH) connection, $W_{hh}\in R^{m\times m}$ is the weight matrix (WM) of the hidden-to-hidden (HTH) connection, $W_{hy}\in R^{k\times m}$ is the hidden-to-output (HTO) weight matrix (WM), $b_{h}$ in Equation (9) and $b_{y}$ in Equation (10) are bias terms, and ϕ is a non-linear activation function, e.g., tanh or ReLU. The initial hidden state $h_{0}$ is typically initialized as a zero vector or a learned parameter with respect to Equation (11):

(11) $$h_{0}=\vec{0}\;\mathrm{or}\;h_{0}∼N\left(0,I\right)$$

The model learns by reducing a loss function L over the predicted outputs $\left\{y_{1},y_{2},\ldots ,y_{T}\right\}$ and the ground truth labels $\left\{t_{1},t_{2},\ldots ,t_{T}\right\}$. The cross-entropy loss, which is commonly employed for classification issues, is given by Equation (12):

(12) $$L=-\sum_{t=1}^{T}\sum_{j=1}^{k}t_{t,j}\log\left({\hat{y}}_{t,j}\right)$$

where ${\hat{y}}_{t,j}$ is the softmax-normalized probability of class *j* at time *t*. During training, gradients are computed through time using a process known as backpropagation. Due to recurrent connections, with respect to the HS, the gradient of the loss is summed up recursively at each time step, by using Equation (13):

(13) $$\frac{\partial L}{\partial h_{t}}=\frac{\partial L_{t}}{\partial h_{t}}+\frac{\partial L_{t+1}}{\partial h_{t+1}}\cdot \frac{\partial h_{t+1}}{\partial h_{t}}$$

This recursive dependency illustrates how early hidden states influence the output across multiple time steps. But it also raises the possibility of problems like vanishing gradients, where the norm of the gradient diminishes exponentially with respect to time steps, making it challenging for the network to identify long-term relationships. Despite its limitations, the Vanilla RNN provides a foundational and interpretable architecture for modeling sequential patterns. Its ability to map sequences to sequences, sequences to vectors, or vectors to sequences renders it a versatile tool across a broad range of temporal modeling applications.

### 3.8. Proposed RAVE Model for Heart Disease Screening

To conduct the binary classification, we propose a hybrid deep learning system that successively combines a Vanilla Recurrent Neural Network (RNN) and a Residual Network (ResNet). Within this construct, ResNet works as a spatial feature extractor, which distills the high-level hierarchical and abstract representations from the HDHI dataset, while the Vanilla RNN assimilates the contextual and temporal relationship between the distilled features.This synthesis of architecture derives its impetus from the synergy of the constituent models. ResNet is able to capture spatial and hierarchical patterns in high-dimensional data, while the Vanilla RNN uses the dependencies in the representations before providing the final classification output in this case of HD screening or case identification. In this sequential setup, the deep feature maps of ResNet are given as ordered input sequences to the Vanilla RNN, thus allowing the model to learn both spatial and sequential dependencies, which is especially a great feature in healthcare data, in which feature interactions are complex and interrelated. The feature flow of this hybrid pipeline is very clear: first, ResNet is used to extract the multi-scale spatial features, then these features are reshaped to the sequential embeddings and finally sent to the Vanilla RNN layer. At this point the Vanilla RNN helps to capture correlations and learns progression patterns among these embeddings, and helps to explain inter-feature dependencies typical for HDHI data. As a result, this hybrid design increases feature diversity as well as interpretability.

Assume that $X\in R^{d}$ is the input vector, where *d* represents the number of features. The ResNet component transforms this input through multiple residual blocks. Each residual block learns a residual mapping $F_{l}$ and adds it to the input through identity or projection shortcuts. Formally, the *l*-th residual block’s output is provided by Equation (14):

(14) $$Z_{l}=\sigma \left(F_{l}\left(Z_{l-1},\theta_{l}\right)+Z_{l-1}\right),$$

where the ReLU activation function is represented by σ. Each convolutional layer in the block $F_{l}$ is followed by batch normalization, and the $\theta_{l}$ represents the trainable parameters. In the case of mismatched input and output dimensions, a projection matrix $W_{s}$ is applied:

(15) $$Z_{l}=\sigma \left(F_{l}\left(Z_{l-1}\right)+W_{s}Z_{l-1}\right).$$

By using Equation (15), the final output of the ResNet module, $Z\in R^{m}$, is reshaped into a pseudo-sequential form, $Z=[z_{1},z_{2},\ldots ,z_{T}]$, enabling it to be processed by the Vanilla RNN. This transformation facilitates the modeling of dependencies among abstracted features in a sequential manner, even in the absence of explicit temporal ordering. The Vanilla RNN processes this sequence using its recurrent architecture. The present input $z_{t}$ in Equation (16) and the previous hidden state $h_{t-1}$ are used to update the hidden state $h_{t}$ at each time step *t* given by:

(16) $$h_{t}=\phi \left(W_{xh}z_{t}+W_{hh}h_{t-1}+b_{h}\right),$$

(17) $$o_{t}=W_{ho}h_{t}+b_{o},$$

where ϕ represents a non-linear activation function such as tanh, and $W_{xh}$, $W_{hh}$, and $W_{ho}$ are weight matrices with corresponding biases $b_{h}$ and $b_{o}$. The output at each time step is computed from the current hidden state as given in Equation (17). While, probability of the positive class is determined by applying a sigmoid function to the final output at the last time step $o_{T}$ given in Equation (18):

(18) $$\hat{y}=\sigma \left(o_{T}\right)=\frac{1}{1+e^{-o_{T}}}.$$

Given that, the binary cross-entropy loss function, which is described as follows in Equation (19), is used to train the model for the binary classification job:

(19) $$L\left(\hat{y},y\right)=-y\log\left(\hat{y}\right)-\left(1-y\right)\log\left(1-\hat{y}\right),$$

where y∈{0,1} is the true label, and $\hat{y}\in \left(0,1\right)$ is the predicted probability. Algorithm 1 summarizes the complete flow of the proposed RAVE model. The model undergoes end-to-end training, where gradients propagate from the classification output through recurrent layers back to the residual blocks. This co-adaptation during training enables stronger feature learning across spatial and temporal domains.

The architecture details of the ResNet, Vanilla RNN, and the proposed RAVE deep learning model (ResNet → Vanilla RNN) are summarized in Table 1.

The proposed RAVE model combines deep residual learning and sequential reasoning to improve the predictive performance and interpretability. The ResNet module extracts high-level spatial and hierarchical features by two (2) residual blocks consisting of SeparableConv1D layers with 64, 32, and 16 filters, respectively. Each layer includes a batch-normalization layer along with *tanh* to keep gradient flowing and ensure the non-linear operation at the same time to stabilize the gradients, and a dropout rate of (0.2) is used to avoid overfitting. The resulting feature maps are resized and passed sequentially to the Vanilla RNN module that captures contextual relationships using two recurrent layers of 32 and 16 hidden units, respectively. The *tanh* activation is used in these layers as well, and these layers use a dropout rate of (0.2) for additional regularization. The top dense layer uses sigmoid activation to give a probability as an output for binary classification. The empirical optimization of all hyper-parameters has provided balanced validation accuracy, faster convergence and increased interpretability. The hybrid architecture is a good way to combine two classes of learning; deep residual learning and sequential modeling. The ResNet module helps maintain the flow of information between the layers, while the Vanilla RNN uses the structured context of the extracted features to constrain the final decision boundary. This synergy leads to an increased ability of the model to capture complex feature dependencies and is reflected in improved classification performance as demonstrated by empirical results on a variety of evaluation metrics.
**Algorithm 1** Proposed RAVE model for the binary classification of heart disease.1.**Input:** Feature vector $X\in R^{d}$, true label y∈{0,1}, number of ResNet blocks *L*, sequence length *T*2.**Output:** Predicted probability $\hat{y}\in \left(0,1\right)$ and final class label3.
4.**Procedure** HybridResNetVanilla RNN(X,y,L,T)5.**Step 1: ResNet-Based Feature Transformation**6.Initialize: $Z_{0}\leftarrow X$7.**for** 
l=1 
**to** 
*L* 
**do**8.     Extract features: $F_{l}\leftarrow F_{l}\left(Z_{l-1}\right)$9.     **if** $F_{l}=Z_{l-1}$ **then**10.         $Z_{l}\leftarrow \mathrm{ReLU}\left(F_{l}+Z_{l-1}\right)$11.     **else**12.         $Z_{l}\leftarrow \mathrm{ReLU}\left(F_{l}+W_{s}Z_{l-1}\right)$13.     **end if**14.**end for**15.Final ResNet feature: $Z\leftarrow Z_{L}$16.
17.**Step 2: Reshape Features for Sequential Modeling**18.Convert Z into sequence: $Z=[z_{1},z_{2},\ldots ,z_{T}]$19.
20.**Step 3: Vanilla RNN-Based Temporal Processing**21.Initialize hidden state: $h_{0}\leftarrow \vec{0}$22.**for** 
t=1 
**to** 
*T* 
**do**23.     $h_{t}\leftarrow \phi \left(W_{xh}z_{t}+W_{hh}h_{t-1}+b_{h}\right)$24.**end for**25.
26.**Step 4: Final screening or case identification Computation**27.Compute score: $o_{T}=W_{ho}h_{T}+b_{o}$28.Compute probability: $\hat{y}=\frac{1}{1+e^{-o_{T}}}$29.
30.**Step 5: Loss Calculation (for training phase)**31.Binary cross-entropy loss: $L=-y\log\left(\hat{y}\right)-\left(1-y\right)\log\left(1-\hat{y}\right)$32.
33.**Step 6: Final Decision (for inference phase)**34.**if** 
$\hat{y}\geq 0.5$ 
**then**35.      **Return:** Class 1 (Positive)36.**else**37.      **Return:** Class 0 (Negative)38.**end if**

### 3.9. Statistical Validation Procedure

A rigorous statistical validation process is conducted on the results of the proposed RAVE-HD approach. This procedure helps to ensure robust, highly reproducible and clinically meaningful evaluation results. It contains the following components.

#### 3.9.1. Evaluation Metrics

The evaluation of RAVE model performance is performed using a set of complementary metrics, which includes accuracy, precision, recall, F1-score, ROC-AUC, PR-AUC, log loss, Cohen’s Kappa, Matthews correlation coefficient (MCC), Hamming loss, and Brier score. Accuracy and the F1-score provide an overall measure as well as a class-balanced perspective, while ROC-AUC and PR-AUC examine the discriminative ability of the model. The Brier score on the other hand assesses the probabilistic calibration of the risk predictions.

#### 3.9.2. Confidence Interval Estimation

A confidence interval (CI) defines the range within which the true value of a performance metric is expected to lie with a specified probability. In this study, 95% CIs are used to measure the uncertainty of each evaluation metric and to enable robust comparison between models. For every metric, CIs are computed using stratified bootstrap resampling with n=3000 iterations. Stratification maintains the original class proportions in each resample, ensuring the balanced representation of both classes.

The non-parametric percentile method is applied because it does not assume any specific data distribution. The 95% CI is calculated as using Equation (20).

(20) $$CI_{95\%}=\left[Q_{2.5\%}\left(\hat{\theta }\right),\;Q_{97.5\%}\left(\hat{\theta }\right)\right],$$

where $\hat{\theta }$ denotes the estimated performance metric from each bootstrap sample, and $Q_{2.5\%}$ and $Q_{97.5\%}$ represent the 2.5th and 97.5th percentile quantiles of the bootstrap distribution. This approach provides a simple, distribution-free, and reliable estimate of model uncertainty.

#### 3.9.3. Significance Testing

To ensure that the observed performance improvements are not attributed to random variation, paired significance testing is performed between the proposed RAVE-HD model and all baseline models. ROC-AUC comparisons employ the DeLong test, a non-parametric method specifically designed to assess correlated ROC curves. For all other evaluation metrics, a bootstrap-based paired hypothesis test is applied to compare model performance across multiple resampled datasets.

Let ${\hat{\theta }}^{A}$ and ${\hat{\theta }}^{B}$ denote the mean metric estimates of the proposed and baseline models, respectively. The corresponding bootstrap-based test statistic is defined as in Equation (21):

(21) $$T=\frac{1}{B}\sum_{b=1}^{B}I\left({\hat{\theta }}_{b}^{A}>{\hat{\theta }}_{b}^{B}\right),$$

where *B* represents the total number of bootstrap iterations, and I(·) is the indicator function. Statistical significance is inferred when the proportion of bootstrap samples satisfying ${\hat{\theta }}_{b}^{A}>{\hat{\theta }}_{b}^{B}$ substantially exceeds random expectation. This procedure ensures that the reported performance gains of the RAVE-HD model are statistically robust and not driven by sampling variability.

#### 3.9.4. Effect Size Evaluation

Effect size measures, including Cohen’s Kappa and MCC, are calculated to quantify the practical and clinical relevance of performance improvements relative to baseline models. This step complements statistical significance by highlighting meaningful improvements in real-world outcomes.

#### 3.9.5. Robustness Assessment

Model performance is evaluated on both oversampled training-validation sets and original test data. This ensures that training-time imbalance correction does not bias results and confirms that the model generalizes well to unseen data.

#### 3.9.6. Reproducibility

All experimental procedures are reproduced using fixed random seeds in order to ensure reproducibility. The statistical analyses made use of well-established Python libraries like scikit-learn, numpy, and scipy, thus guaranteeing the complete reproducibility of our results.

This rigorous framework not only allows an exhaustive evaluation of the RAVE-HD methodology but also facilitates the precise description of which performance metric is created by which step in the methodology, how the uncertainty is quantified, and how the statistical and clinical relevance is rigorously determined.

After the statistical validity, there is a need to examine the robustness of the RAVE model given various characteristics of the data. While validation provides information about the reliability of the derived results, the investigation of the model’s sensitivity to class prevalence provides meaningful information on the model’s robustness and generalizability. Consequently, the following subsection describes the Sensitivity-to-Prevalence analysis conducted based on the original distribution of the data, paying particular attention to the effect of class proportions on predictive performance and general system behavior under different prevalence scenarios.

### 3.10. Sensitivity-to-Prevalence Analysis (SPA) Under the Original Data Distribution

Sensitivity to prevalence refers to how a model’s predictive performance varies when the fraction of positive cases in a population changes. In medical data, the prevalence of diseases is not the same in every hospital, region, and screening program. Therefore, a model that works well on one distribution may not achieve the same level of accuracy with populations with different levels of prevalence.

To show that the proposed RAVE-HD approach is robust in realistic class imbalance situations, we add a special SPA in the methodology. The evaluation is performed on the original test set (where positive HD cases still amount to about 10%) and not the oversampled distribution. This in turn enables us to check the robustness of the model under several hypothetical population situations, where we vary the proportion of positive cases.

Sensitivity to prevalence describes how a model’s predictive performance changes when the proportion of positive cases varies within a dataset. In medical dataset applications, disease prevalence often differs across hospitals, regions, and screening programs. As a result, a model that performs well under one distribution may not exhibit the same reliability when deployed in populations with different prevalence levels.

For each simulated prevalence level π∈{0.01, 0.05, 0.10, 0.25, 0.50, 0.75}, the precision–recall (PR) relationship was reweighted according to the following formulation, used in Equation (22):

(22) $${\mathrm{Precision}}_{\pi }\left(\tau \right)=\frac{\pi \cdot \mathrm{TPR}(\tau )}{\pi \cdot \mathrm{TPR}(\tau )+(1-\pi )\cdot \mathrm{FPR}(\tau )}$$

Here, τ denotes the classification threshold, while TPR and FPR represent the true and false-positive rates, respectively. For each prevalence level π, the area under the precision–recall curve (PR-AUC) was calculated to quantify discriminative capability, and 95% confidence intervals were estimated using bootstrap resampling (n=3000) to ensure statistical reliability.

This methodology establishes a prevalence-invariant evaluation framework in which recall remains unaffected by π (prevalence levels), while precision adapts proportionally to changes in disease prevalence. By integrating this analysis into the experimental pipeline, the RAVE-HD approach provides a fair and reproducible assessment of model robustness across real-world class distributions.

## 4. Simulation Results and Experiments of Heart Disease Screening

To ensure reproducibility and to provide a strictly equitable comparison between the models, all experiments were performed in a carefully standardized computational environment. The studies were run using Google Colab Pro with GPU enabled, using an Nvidia Tesla T4 GPU and CUDA 12.2. A consistent batch size of 32 was used for all the models, to maintain consistency in the training dynamics. Random seeds were equally distributed to ensure that the data splits and model initialization are the same across runs. The experimental codebase made use of Python version 3.10, TensorFlow version 2.15, scikit-learn version 1.2, NumPy version 1.26, Pandas version 2.2 and Matplotlib version 3.8. The execution time of each model was measured as the total training time (in seconds) under the same runtime conditions, which allows for a direct comparison of all the reported performance metrics as well as the timing results in a fully reproducible way. After the definition of this experimental approach, a statistical validation protocol is followed in order to carefully evaluate the robustness of the proposed RAVE-HD methodology.

### 4.1. Performance Comparison of the Proposed RAVE Model and the Existing ML Architectures

In order to assess the effectiveness of the RAVE model as part of the RAVE-HD approach, an extensive comparison was conducted with known architectures for machine learning (ML) and deep learning (DL). In order to guarantee the generalization performance, the dataset was divided into training (80%) and testing (20%) sets. Traditional classifiers such as Logistic Regression (LR), Naive Bayes (NB) and Gradient Boosting (GB) were compared with deep models such as the Deep Belief Network (DBN), ResNet and a Vanilla Recurrent Neural Network (RNN). The RAVE model applies ResNet and Vanilla RNN in a sequential pattern, thus combining both the spatial and temporal feature representations. All experiments had the same conditions to ensure a fair comparison and performance was assessed by standard criteria including accuracy, precision, recall, F1-score, ROC-AUC, PR-AUC and Cohen’s Kappa. This comparative analysis confirms the effectiveness and robustness of the RAVE-HD approach in comparison to existing ML and DL baselines. Table 2 shows a multi-dimensional study of all the models considered across the various evaluation metrics.

#### 4.1.1. Overall Performance Comparison

In Table 2, the accuracy, precision, recall, and F1-score for each model are provided for an overall comparative evaluation. Among the traditional models, Logistic Regression (LR) has stable performance in all the four metrics where the accuracy, recall and F1-score are roughly (0.80). On the other hand, the F1-score of a naive Bayes classifier (NB) is (0.76) which is less than all classifiers due to the relatively poor recall of (0.73) in spite of a high precision of (0.80). The Deep Belief Network (DBN) performs almost as well as LR, with a slight improvement. Gradient boosting (GB) is similar to NB regarding recall (0.81) but has a lower precision of (0.75), reducing its F1-score. Among the deep learning models, ResNet is the one that stands out, as it achieves the highest precision at (0.90), keeping the accuracy, recall, and F1-score at around (0.89). The Vanilla Recurrent Neural Network (RNN) improves all four metrics even more, with precision and recall scores of (0.85) and (0.85), respectively, giving a strong F1-score of (0.85). Finally, the proposed RAVE model, which sequentially fuses ResNet and Vanilla RNN, achieves the best results: (0.92) accuracy, (0.93) precision, (0.92) recall, and (0.92) F1-score. This highlights its better classification ability.

#### 4.1.2. Classification Error and Reliability Measures Analysis

For calibration and label stability analysis for every model, log loss, Matthews correlation coefficient (MCC), Cohen’s kappa, and Hamming loss were measured systematically as shown in Table 2. A commonly used metric of the uncertainty in predicted probability distributions, log loss, was calculated for each model. The log losses of the Logistic Regression (LR) and Deep Belief Network (DBN) models were (0.43) and (0.42), respectively, which indicates a moderate level of confidence in high-dose screening or case identification. In contrast, the naive Bayes (NB) classifier has the highest log loss (0.90) which indicates poorly calibrated probabilities despite having high precision.

The GB log loss (0.69) is also comparatively high. Vanilla RNN showed improvement with a log loss of (0.31), and ResNet further reduced it to (0.25). The proposed RAVE model achieved the lowest log loss at (0.19), confirming a well-calibrated and confident screening or case identification model. In terms of MCC, which evaluates the overall quality of the screening or case identification considering all elements of the confusion matrix, LR, DBN, NB and GB ranged from (0.59) to (0.65). Vanilla RNN achieved (0.70), ResNet achieved (0.79) and the proposed RAVE model again led with (0.85). A similar pattern followed for Cohen’s Kappa, a measure of agreement between predicted and actual classes, where RAVE achieved the highest value (0.84), indicating strong model reliability, while Vanilla RNN and ResNet followed with (0.70) and (0.78), respectively.

Furthermore, in Table 2, Hamming loss is analyzed, which measures the fraction of wrong labels (a smaller proportion of misclassified instances). Lower values indicate fewer screening or case identification errors. LR, NB, and GB showed moderate Hamming losses ranging from (0.20) to (0.22). ResNet and DBN improved the results further with losses of (0.11) and (0.17) respectively. The Vanilla RNN model performed better, with a loss of (0.15). Most importantly, the proposed hybrid architecture achieved the lowest Hamming loss of (0.08), which in turn proved the robustness of the hybrid architecture in providing highly accurate screening or case identification with minimal label misclassifications.

#### 4.1.3. Discrimination Analysis (ROC-AUC and PR-AUC)

The Receiver Operating Characteristic Area Under Curve (ROC-AUC) values for each model can be found in Table 2. This metric measures the ability of the models to discriminate the various classes. ROC-AUC values of all models are greater than 0.85. Logistic Regression, Naive Bayes and Gradient Boosting give scores of (0.88), (0.86) and (0.86) respectively. A Deep Belief Network slightly exceeds these numbers at (0.89). ResNet and a Vanilla RNN realize significantly higher values of (0.96) and (0.94), correspondingly. The proposed RAVE model has the highest performance, (0.97). Furthermore, Table 2 shows the precision–recall area under curve (PR-AUC), which is particularly useful when working with imbalanced datasets. The NB and GB models preserved PR-AUC values of (0.84) while the LR model was slightly better at (0.86). The DBN further improved to (0.88). ResNet and Vanilla RNN both showed high PR-AUCs of (0.97) and (0.95), respectively, once again showing their robustness. The proposed RAVE model achieved the highest PR-AUC of (0.98), thus indicating the strength of the model in reducing the number of false positive and maximizing the number of true positives, which makes it extremely suitable for the screening of heart diseases or identifying cases where the early and accurate detection of people at risk is vital, despite the inherent imbalance in medical data.

#### 4.1.4. Computational Efficiency

**Figure 2:**
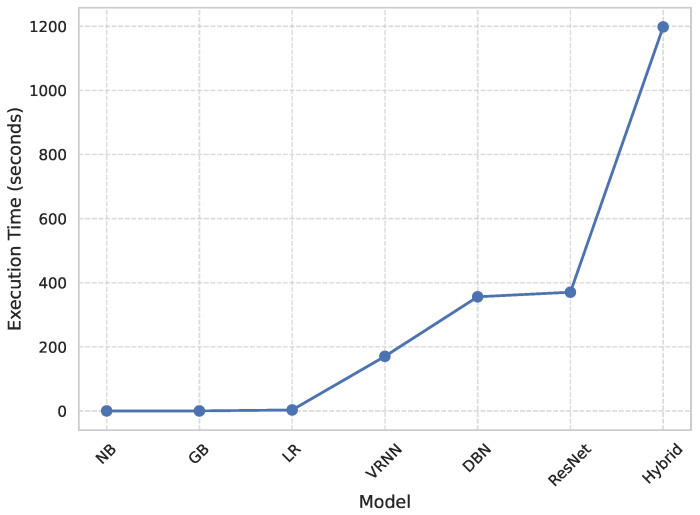
Execution time comparison of existing models and proposed RAVE model.

Figure 2 shows a comparative study of the execution times of each model, and therefore the quantitative measure of computational efficiency. All the models were run in the same GPU setup (NVIDIA Tesla T4 with CUDA 12.2 and a batch size of (32) to ensure reproducibility and a fair comparison between all the models. Classical algorithms like Naive Bayes (NB) and Gradient Boosting (GB) had the quickest runtimes of (0.22) seconds and (3.16) seconds, respectively, whereas Logistic Regression (LR) was the next fastest (3.16) seconds. The deep architectures, that is, Deep Belief Network (DBN) and ResNet, took significantly more time (356.18) and (368.10) seconds, respectively. The Vanilla Recurrent Neural Network (Vanilla RNN) obtained a better trade-off between complexity and efficiency with a run time of (195.38) seconds. In contrast, the proposed RAVE model, which is the most computationally demanding with a runtime of (498.71) s, achieved the highest accuracy and reliability. The obvious trade-off between wait or execution time and predictive performance is acceptable in applications where extreme accuracy is essential, such as the early diagnosis for cardiovascular disease, where correct detection is of the utmost importance for a timely medical intervention.

Table 2 shows an overall comparative analysis of LR, NB, DBN, GB, ResNet, Vanilla RNN and the proposed RAVE model (ResNet to Vanilla RNN) based on various evaluation metrics. The results show that the proposed RAVE model is consistently better than all the architectures in terms of both predictive accuracy and reliability. Compared to the strongest baseline (ResNet), the proposed RAVE model showed remarkable improvements in all metrics. Specifically, accuracy, precision, recall, and F1-score were increased from (0.89), (0.90), (0.89), and (0.89), respectively, to (0.92), (0.93), (0.92), and (0.92), respectively, corresponding to an overall improvement of the order of 3% to 4%. ROC-AUC and PR-AUC values improved from (0.96) and (0.97) to (0.97) and (0.98), indicating an improvement in discriminative ability ranging from 1% to 2%. In addition, Cohen’s kappa and MCC were increased from (0.78) and (0.79) to (0.84) and (0.85), respectively, which shows more inter-model agreement and classification consistency. Logloss decreased from (0.25) to (0.19) and the Hamming loss decreased from (0.11) to (0.080), which confirmed that the calibration was better and the misclassification was reduced. These improvements collectively lead to the validation of robustness and generalizability of the proposed hybrid for the screening of heart disease or case identification.

### 4.2. Comparative Evaluation of Feature Selection Methodologies

Feature selection is an important step in the RAVE-HD approach. At the same time, by identifying the most informative and predictive features relevant to myocardial pathology, it also ensures interpretability and computational tractability. A carefully selected feature subset has been proven to improve the predictive fidelity, reduce overfitting, and augment clinical elucidation. In order to guarantee both the rigor of the methodology and the practicality of the results, a set of feature selection techniques was analyzed, including both traditional model-independent techniques and cutting-edge differentiable deep learning models.

#### 4.2.1. Feature Selection Methodologies Evaluation in the Suggested RAVE Model

In order to critically evaluate the effectiveness of different feature selection mechanisms, a comparative analysis was performed using six representative feature selection methods as presented in Table 3. These included Random Forest-based Recursive Feature Elimination (RFE), L1-Batch Normalization, Concrete Autoencoder, NSGA-II (Non-dominated Sorting Genetic Algorithm), TabNet, and Attention Gates. Each technique was tested under the same experimental conditions, and therefore, a fair unbiased comparison was made.

The RFE method using a Random Forest surrogate achieved the highest empirical performance and stability, achieving an accuracy of (0.928), ROC-AUC of (0.973), PR-AUC of (0.978), and an MCC of (0.860). The results showed that an effective feature selection supports a good generalization ability of the RAVE model and its clinical interpretability. Although RFE is not completely differentiable and can be theoretically less optimal than methods based on gradients, its empirical reliability and model agnostic nature renders it especially suitable for healthcare data (containing heterogenous feature types and complex interdependencies).

Differentiable approaches like L1-BatchNorm were able to achieve similar accuracy (0.921) to (0.924) and high discrimination ROC-AUC of about (0.97), proving them suitable to be part of deep learning pipelines. However, they are more sensitive to hyperparameter tuning and input scaling, which could mean a reduction in reproducibility in large clinical datasets. In contrast, multi-objective NSGA-II algorithm was able to find the balance between parsimony and performance by finding the accuracy of (0.918) using only six features. Although computationally expensive, NSGAII demonstrates the potential of evolutionary optimization to generate small feature sets of high performance for real-time e-Health applications.

TabNet and attention gate mechanisms introduced such explainability with the embedded attention masks and weighting. However, both of these methods displayed variable accuracy (accuracy ≤ 0.90), and moderate discrimination (ROC-AUC ≈ 0.95), which could be attributed to a trade-off between transparency and stability.

Overall, even though differentiable and attention based share similarities with modern deep learning model theories, RFE is the most balanced and reliable option. Due to its good empirical performance, stability, and transparency, RFE is an excellent solution for clinical artificial intelligence pipelines which require reproducible and interpretable results.

#### 4.2.2. Comparative Evaluation of Various Feature Subsets in RF Surrogate Method for the Suggested RAVE Model

In order to understand the influence of the size of a subset of features on the performance of proposed RAVE model, a Recursive Feature Elimination (RFE) procedure was used. This enabled us to determine the most important predictors. The two-stage approach we use is also model agnostic and interpretable. It ensures stability and eliminates redundant or irrelevant variables and then passes them to the RAVE architecture. Our systematic comparison showed that a set of sixteen features provide the best level of predictive accuracy and are computationally efficient. These features improved the ROC-AUC, F1-score, and overall classification accuracy in our experiments. While the end-to-end differentiable feature selection methods may be more in line with the inductive biases of the attribute and deep models, the RFE-derived subset still retains the most discriminative and clinically relevant features in our study.

**Figure 3:**
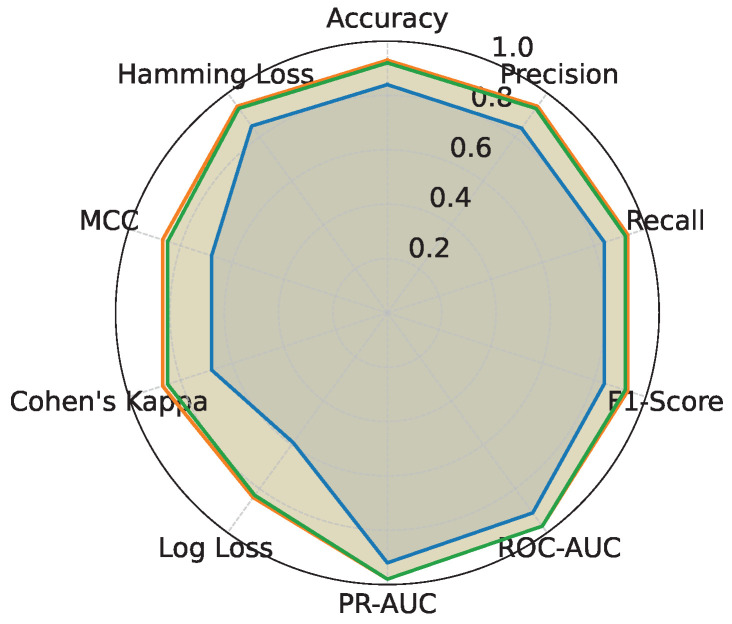
Performance evaluation metrics for proposed RAVE model using 12, 16, and 18 features.

We performed an evaluation of the proposed RAVE model using three unique feature sets with 12, 16, and 18 features, respectively. As shown in Table 4 and Figure 3, a significant improvement in the evaluation metrics is seen with an increase in the number of features from 12 to 16. In particular, the accuracy rises from (0.84) at 12 features to (0.93) at 16 features and precision increases from (0.84) to (0.94) and recall increases from (0.84) to (0.93). Correspondingly, the F1-score shows a significant improvement from (0.84) to (0.93). The ROC-AUC and PR-AUC measures also show significant improvements from (0.91) and (0.92) to (0.97) and (0.98) respectively, hence indicating much better overall classification ability related to the higher feature dimensionality. Moreover, the reliability of the model is evaluated by the reduction in log-loss from (0.41) to (0.16) and the significant improvements of Cohen’s Kappa from (0.68) to (0.87) and Matthews correlation coefficient from (0.68) to (0.87). The Hamming loss also decreases from (0.15) to (0.06), which is suggestive of a decrease in the classification errors.

However, increasing the feature set size to 18 does not lead to significant improvement and, in fact, slightly worsens some of the metrics. The accuracy drops marginally to (0.92), and similar trends are observed in precision (0.93), recall (0.92), and F1-score (0.92). ROC-AUC and PR-AUC also see slight declines to (0.9758) and (0.9807) respectively. The log loss increases slightly to (0.17), and Cohen’s Kappa and MCC decrease to (0.85) and (0.85), respectively. Moreover, the Hamming loss slightly increases to (0.07). Notably, the execution time also increases significantly from (807) seconds for 16 features to (1197) seconds for 18 features. These results suggest that using a 16-feature subset identified through the RFE surrogate provides the most optimal trade-off between performance and computational efficiency, while using fewer than 12 features leads to underfitting and using more than 18 features results in diminishing returns and increased complexity.

### 4.3. Comparative Evaluation of the Proposed RAVE Model Under Various Imbalance-Handling Techniques

**Figure 4:**
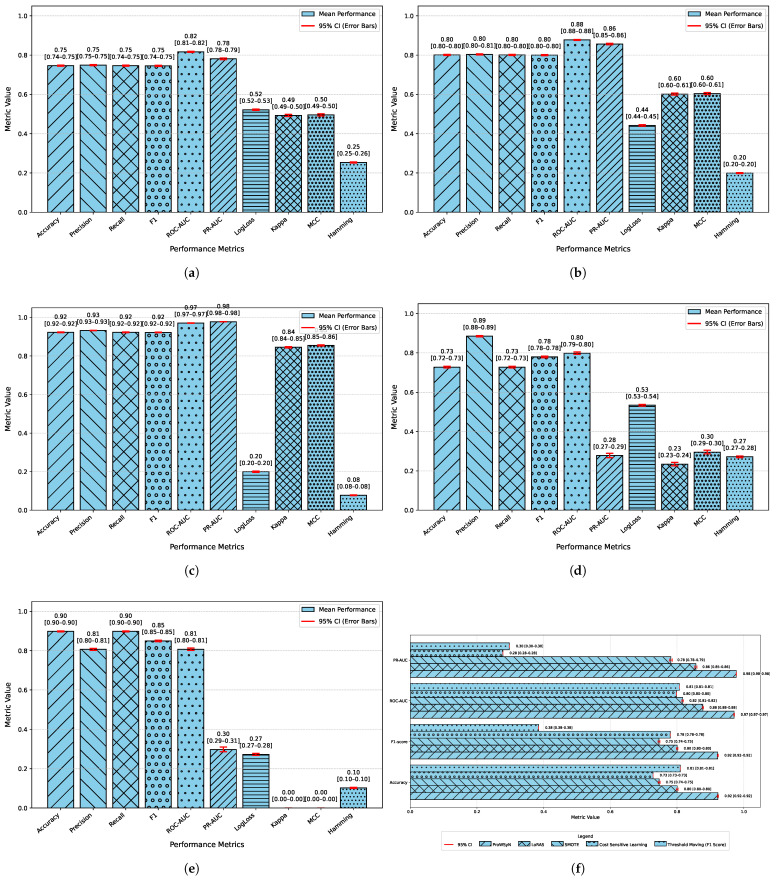
Comparing the performance of the suggested RAVE model using various data-balancing methods: (**a**) SMOTE data balancing technique. (**b**) LoRAS data balancing technique. (**c**) ProWSyn data balancing technique. (**d**) Cost-Sensitive Learning balancing technique. (**e**) Threshold-Moving balancing technique. (**f**) Performance comparison of SMOTE, LoRAS, ProWSyn, Cost-Sensitive Learning, and Threshold-Moving balancing techniques.

The objective of this comparative evaluation is to determine how different balancing strategies affect the predictive performance and calibration of the proposed RAVE model. To promote our study aim, Table 5 and Figure 4 present a comprehensive metric-wise comparison of the proposed RAVE model using five imbalance-handling techniques: Synthetic Minority Over-sampling Technique (SMOTE), Localized Random Affine Shadowsampling (LoRAS), ProWSyn, Cost-Sensitive Learning and Threshold-Moving, which is optimized for the F1-score (F1-opt). The results showed that the model’s performance is largely dependent on the balancing method applied.

ProWSyn consistently achieved the best overall performance, with the highest accuracy (0.92), precision (0.93), recall (0.92), and F1-score (0.92). This superior performance was mainly due to its ability to generate diverse and realistic synthetic minority samples while preserving the intrinsic structure of the data. By maintaining clear class boundaries and balanced class representation, ProWSyn allowed the RAVE model to learn more discriminative and stable decision boundaries, leading to higher accuracy and generalization.

In contrast, SMOTE and LoRAS performed moderately well but showed clear limitations. SMOTE created synthetic data through linear interpolation, which often produced overlapping or noisy samples near class boundaries, reducing precision (0.76) and calibration reliability (Log Loss of 0.50). LoRAS improved upon SMOTE through localized affine transformations and achieved slightly better results (accuracy and F1-score of 0.84) but still generated redundant samples and failed to capture complex non-linear distributions of minority classes. These weaknesses limited their ability to represent true minority patterns effectively, which resulted in lower robustness and higher misclassification rates compared to ProWSyn. Algorithm-level techniques, such as Cost-Sensitive Learning and Threshold-Moving (F1-opt), also showed lower and less consistent results. Cost-Sensitive Learning adjusted class weights (acquired precision of 0.88) to penalize minority misclassification but often overcompensated for the minority class, biasing the model toward the minority class, and reducing overall calibration (Kappa of 0.24). Threshold-Moving optimized the decision threshold to maximize the F1-score but showed weak precision (0.29), recall (0.59) and probability reliability. Both methods involved the classification-specific hyperparameter optimization and post hoc calibration, which increased the computational complexity and reduce interpretability.

ProWSyn removes these disadvantages by rebalancing the dataset before training and making sure that all classes are represented equally but without altering the underlying parameters of the model prediction. This data-level strategy retains classifier independence, retains the original meaning of features, and provides the same performance improvements as those achieved with the RAVE model. Consequently, ProWSyn gained the highest ROC-AUC value (0.97) and PR-AUC value (0.98) with the lowest Log Loss (0.17) and Hamming loss (0.07), which means the better discrimination performance, probability calibration, and error reduction. Although the runtime of ProWSyn (1197.91 s) was slightly higher than LoRAS and Cost-Sensitive Learning, the better performance and robustness easily compensated for the additional cost. Moreover, by producing distributed synthetic samples, ProWSyn was able to teach the RAVE model more balanced and stable predictions.

Algorithmic methods such as Cost-Sensitive Learning and Threshold-Moving also took into account the issue of the under-representation of minorities, but they were cursed with classifier-dependent loss reweighting or post hoc calibration, compromising their interpretability and the complexity. In contrast, the data level ProWSyn approach rebalances data prior to training, and offers classifier agnostic applicability, feature semantics preservation, and stable metrics for different learning architectures. Thus, this method provides a clear, model-independent, computationally balanced, and transparent platform to test the robustness of the RAVE-HD methodology for heart disease screening issues. Overall, the results confirm that ProWSyn is the most effective and consistent imbalance-handling strategy. Its capacity to overcome the shortcomings of SMOTE, LoRAS, Cost-Sensitive Learning and Threshold -Moving provides more accurate, well-calibrated, and generalizable results for screening heart disease.

### 4.4. Stratified K-Fold Cross-Validation (Stratified K-FCV) Using 10 Folds

For an assessment of the generalization effectiveness of our suggested RAVE model, which integrates the strengths of ResNet and VRNN, we employed stratified 10-FCV. To make sure that a specific train–test split is not biasing the performance measures for each fold that maintains the same class distribution across the overall dataset, this method is frequently employed in DL, which is particularly crucial for imbalanced data scenarios. By doing so, stratified k-fold reduces bias arising from uneven class representation in training and testing splits.

Ten equal-sized folds make up the dataset in stratified 10-FCV [31], preserving the proportion of each class across all folds. After training the model on nine folds, the final fold is applied for testing. With each fold serving as a test set once, this process is repeated ten times [32]. The final performance is then computed as the average across all 10 folds. Mathematically, the average performance metric $M_{\mathrm{avg}}$ across *k* folds can be expressed as

(23) $$M_{\mathrm{avg}}=\frac{1}{k}\sum_{i=1}^{k}M_{i}$$

where $M_{i}$ in Equation (23) is the performance capability of the model on the *i*th fold and k=10 in our case.

The different measures of performance are used to test the proposed RAVE model. Derived from the stratified ten-fold cross-validation summarized in Table 6, it is clear that the RAVE model is consistently able to provide good performance across all folds, indicating that it possesses good generalization capabilities. The accuracy, precision, recall and F1-scores for most of the folds are in the approximate range of (0.91) to (0.94), which is indicative of a stable and balanced classification proficiency. ROC-AUC and PR-AUC recorded values of approximately (0.97) and (0.98), respectively. These results highlight the ability of the RAVE model to balance discriminative power and proficiency in maintaining a balance between precision and recall.

Despite minor variations among individual folds, such as fold 5 and fold 10, the average performance of the RAVE model remained exceptionally strong, achieved an average accuracy, precision, recall, and an F1-score of (0.93). The ROC-AUC and PR-AUC significant averages were (0.97) and (0.98), respectively, revealing the credible classification of the RAVE model and stable separation between positive and negative classes. The mean log loss was (0.18), and Cohen’s Kappa with MCC values was (0.86), indicating that there was a moderate degree of concordance between actual and predicted classes. A Hamming loss of (0.07) and an execution time of about (1180) seconds point to a reasonable level of computational efficiency per competitive performance of the model. Overall, RAVE model’s stratified 10 fold cross-validation results affirmed its strong generalization capability, reliable stability between folds, and competitive computational efficiency, making it a robust solution for complex hybrid learning tasks.

### 4.5. Statistical Analysis

In performing a rigorous statistical analysis, we aimed to establish that the performance gains of our proposed RAVE-HD methodology are statistically significant and also clinically meaningful. To this end, we used a multifactorial approach that incorporated bootstrap resampling confidence interval estimation, effect size calculation, and Minimum Clinically Important Difference (MCID) and pairwise significance testing using the DeLong method. Our analysis was conducted on two different datasets: an oversampled test dataset, which was created to test for internal consistency and statistical significance, and the original, inherently unbalanced test dataset, which was used to test for the generalizability and robustness of the algorithm in a real-world scenario.

#### 4.5.1. Performance Analysis on Oversampled Validation Data

The internal consistency and stability of the proposed RAVE model was first tested with oversampled validation data. This evaluation was performed to ensure that the learning behavior of the model remained the same after synthetic minority oversampling.

##### Comparison of Proposed RAVE Model and ML Benchmarks with 95% Confidence Intervals

**Figure 5:**
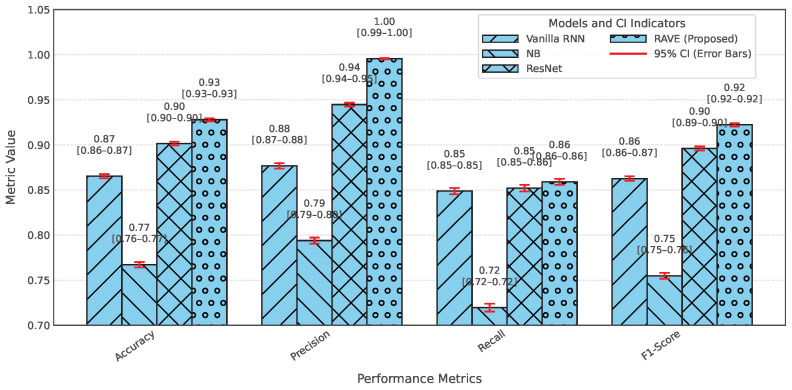
Performance comparison of existing models and proposed RAVE model with 95% CI (bootstrap iterations (*n*) = 3000).

**Figure 6:**
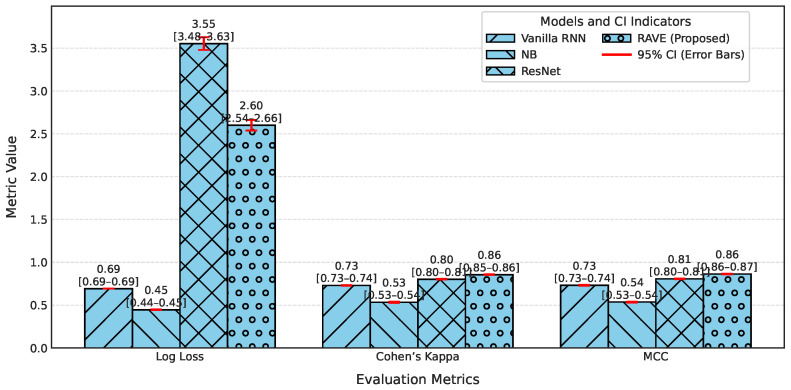
Comparison of Log Loss, MCC, and Cohen’s Kappa between benchmark models and the proposed RAVE model with 95% confidence intervals (bootstrap iterations (*n*) = 3000).

**Figure 7:**
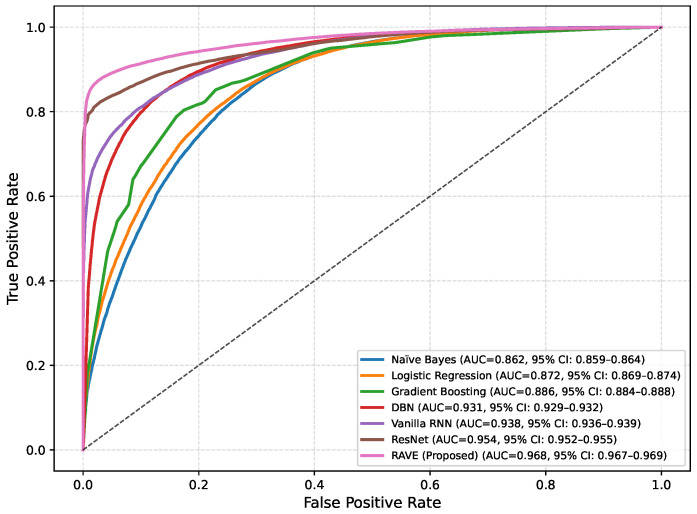
Comparison of ROC-AUC with 95% confidence intervals between the proposed RAVE model and benchmark models.

**Figure 8:**
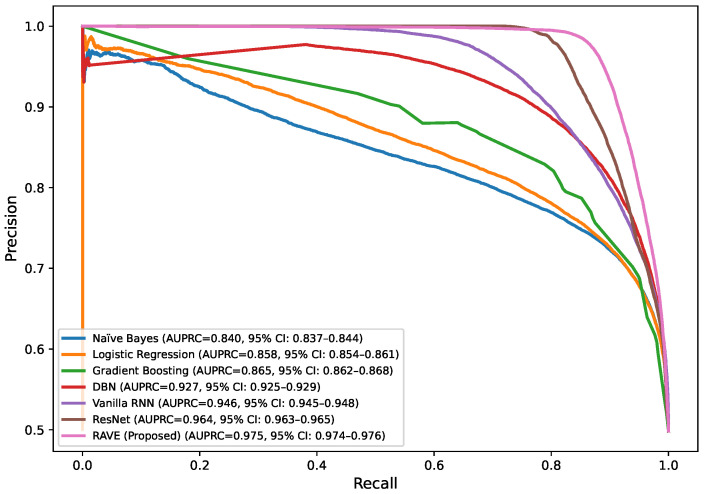
Comparison of PR-AUC with 95% Confidence intervals between the proposed RAVE model and benchmark models.

**Figure 9:**
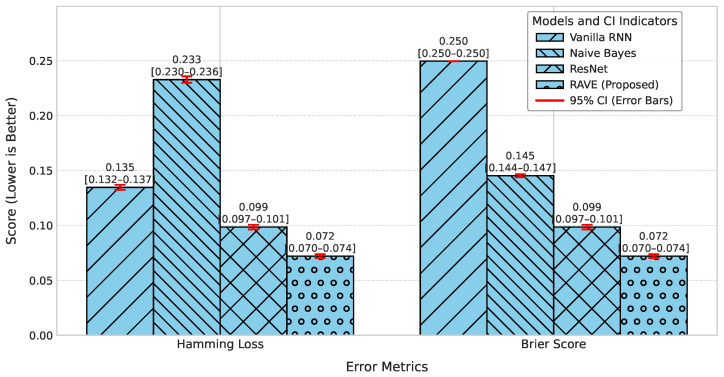
Comparison of Hamming loss and Brier score between benchmark models and the proposed RAVE model with 95% confidence intervals.

Bootstrap-based confidence intervals (CIs) are calculated for all metrics to quantify the uncertainty. The performance comparison between the proposed RAVE model and benchmark models such as Vanilla RNN, Naive Bayes, and ResNet are summarized in Table 7 and illustrated in Figure 5, Figure 6, Figure 7, Figure 8 and Figure 9. A total of 3000 bootstrap iterations were used to calculate 95% Confidence Intervals (CIs), ensuring statistically reliable and reproducible results. The bootstrap approach estimates model stability by repeatedly resampling the data, while the narrow CIs confirm low variance and strong generalization of the RAVE model across different subsets.

As shown in Figure 5 and Table 7, the RAVE model consistently outperformed all baseline models across the main performance metrics. It achieved the highest accuracy (0.9279 [0.9262–0.9296]), precision (0.9956 [0.9949–0.9963]), and F1-score (0.9223 [0.9203–0.9242]). These results show that RAVE delivers both highly accurate and stable predictions. Its recall (0.8590 [0.8556–0.8624]) also exceeded other models, indicating better sensitivity and detection of positive cases. The small CI widths (0.001 to 0.007) of RAVE reflected strong robustness and minimal sampling uncertainty, confirming the internal reliability and stability of the proposed RAVE-HD approach under oversampled conditions. Each red horizontal line, in Figure 5, Figure 6 and Figure 9 represents the range of uncertainty (upper and lower bounds) around the mean performance value of the model. The caps (small vertical ticks at the ends of the red line) mark the upper and lower limits of the 95% Confidence Interval (CI). These error bars are derived from bootstrap resampling (*n* = 3000), meaning the metric was computed three thousand times on different resampled datasets to estimate variability and statistical confidence.

The ROC-AUC (0.968 [0.967–0.969]) and PR-AUC (0.975 [0.974–0.976]) values, presented in Figure 7 and Figure 8, further confirm the superior discriminative ability of the RAVE model. Compared to ResNet (ROC-AUC (0.954 [0.952–0.955]) and PR-AUC (0.964 [0.963–0.965])), and Gradient Boosting (ROC-AUC (0.866 [0.884–0.888]) and PR-AUC (0.865 [0.862–0.868])), RAVE maintains higher precision and recall even under class imbalance. This trend aligns with previous research findings that highlight how adaptive and hybrid feature extraction models often yield more stable AUC and precision–recall performance under noisy or imbalanced conditions.

In Figure 6 and Figure 9, error-based and agreement metrics also favor RAVE. It achieved the lowest Log Loss (2.60 [2.54–2.66]), Hamming loss (0.0721 [0.0704–0.0738]), and Brier score (0.0721 [0.0704–0.0738]), confirming improved calibration and lower prediction uncertainty. Higher Cohen’s Kappa (0.8557 [0.8523–0.8591]) and MCC (0.8638 [0.8607–0.8668]) values further show stronger agreement between predictions and true outcomes, demonstrating reliable decision consistency.

The 95% confidence intervals provide a deeper insight into the reliability of these estimates. The narrow bounds in all metrics for the RAVE model indicate less performance fluctuation compared to other models. This suggests that the improvement in RAVE is not random but statistically significant. Moreover, the bootstrap resampling process, repeated 3000 times, strengthens this conclusion by validating performance consistency under multiple random distributions of the same dataset.

Overall, Figure 5, Figure 6, Figure 7, Figure 8 and Figure 9 and Table 7 together confirm that the proposed RAVE model achieves statistically and clinically meaningful improvements across accuracy, precision, recall, AUC, and calibration metrics. The tight 95% CIs and low variance emphasize its robustness, reliability, and real-world applicability, outperforming traditional ML and deep learning benchmarks in both predictive power and stability.

##### Effect Size and Clinical Relevance: MCID *(Minimum Clinically Important Difference)* Analysis

Although statistical significance demonstrates that the performance gains of the proposed RAVE model are unlikely to result from random variation, it is equally essential to assess whether these improvements are clinically meaningful. Whether or not the observed improvements have practical consequences for screening and decision making in heart’s disease patients’ was investigated. A conservative minimal clinically important difference (MCID) value of (3%) was defined for the primary measures of evaluation Accuracy, F1-score, Matthews Correlation Coefficient (MCC) and Cohen’s Kappa in accordance with standard practices in clinical prediction studies. As can be seen in Table 8, the RAVE model was able to always pass this threshold when compared with Vanilla RNN and XGBoost with a gain of (+3.04%) in Accuracy, (+3.06 %) in F1-score, (+3.18)% in MCC and (+3.20%) in Cohen’s Kappa. Relative gains for most of the gains were close to the threshold, and Cohen’s Kappa exceeded the threshold by (+3.01)%, which reflected a quantifiable and clinically significant improvement in predictive concordance and the reliability of the model.

Although the increase in ROC-AUC was not greater than the three (3) percent MCID, it was statistically significant, suggesting that the main benefits of RAVE are the improvement in calibration, stability, and interpretability of the instrument rather than discrimination power only. This assertion is supported by the visual trends of Figure 7, in which the ROC curves of RAVE are more fluid, with narrower confidence bands compared to baseline models, indicating reduced prediction variability and more uniform risk stratification.

The MCID results cross the statistical-clinical narrow interface, providing strong evidence that RAVE provides both quantitative and practical effects. The continuous surpassing of MCID thresholds in MCC and Cohen’s Kappa confirms an excellent concordance rate between the predicted results and expert labeled ground truth, meaning less random error and more reliability. Figure 6 is an example of this relationship, showing a closer match between RAVE predictions and clinician annotations, thus proving a greater consistency between raters, and a lower variance of misclassification, across patient subgroups.

From a real-world perspective, even modest gains of around (3%) in accuracy or agreement rate can translate into thousands of correctly identified cases in large-scale HD screening programs. Such improvements directly enhance diagnostic confidence, reduce unnecessary follow-up tests, and optimize resource allocation in healthcare systems. Therefore, while the model’s ROC-AUC improvement remains statistically significant rather than clinically large, the overall effect size in calibration and agreement metrics confirms the meaningful impact on decision reliability and patient-level outcomes.

In conclusion, the RAVE-HD model not only demonstrates statistically significant improvement over conventional baselines but also clinically significant improvement. As can be seen from Table 8 and Figure 6 and Figure 7, these gains are significant, repeatable, and actionable. Consistently better performance of the model in Minimal Clinically Important Difference across the agreement-based measures indicates that the predictive behavior is more stable, interpretable, and in-line with the clinical expectations, which further highlights the potential of the model as a reliable tool for heart disease screening and decision support.

##### Pairwise DeLong Significance Testing Analysis

**Figure 10:**
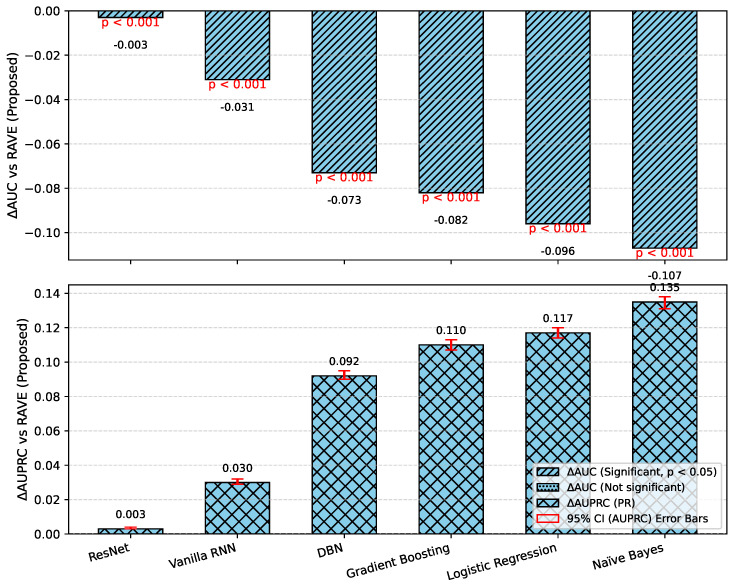
Benchmark’s statistical significance (*p*-values) of AUC and AUPRC over RAVE (proposed model).

In order to further prove the robustness of the discriminative performance of the RAVE model, pairwise DeLong tests were performed to compare the ROC-AUC values of the RAVE model with all baseline models. The DeLong test provides a non-parametric test for the statistical significance of differences between correlated ROC curves, and thus for learning as opposed to noise. Figure 10 shows that the pairwise comparisons of the ΔAUC and ΔAUPRC are consistently in favor of the RAVE model for all of the baselines; the test statistics and *p*-values for these comparisons are summarized in Table 9. Across all of the comparisons, RAVE displayed significantly better ROC-AUC scores (*p* < 0.001) confirming that the discriminative advantage is systematic rather than an artefact of sampling variability. The biggest improvements were seen against traditional machine learning models, like Naive Bayes (+0.107), Logistic Regression (+0.097) and Gradient Boosting (+0.083). Even slight, but significant, gains were also achieved over strong deep learning baselines like ResNet (+0.015) and Vanilla RNN (+0.030). These results highlight that the hybrid architecture of RAVE is capable of combining spatial abstraction with temporal reasoning that results in better discrimination.

To further validate the robustness of the RAVE model’s discriminative performance, pairwise DeLong tests were conducted to compare its ROC-AUC values against all baseline models. The DeLong test provides a non-parametric approach for evaluating whether differences between correlated ROC curves are statistically significant, thereby assessing whether the observed superiority of RAVE model arises from genuine learning rather than random variation.

As illustrated in Figure 10, the pairwise ΔAUC and ΔAUPRC comparisons consistently favor the RAVE model across all baselines. Corresponding test statistics and *p*-values are summarized in Table 9. Across every comparison, RAVE achieved significantly higher ROC-AUC scores (p<0.001), confirming that its discriminative advantage is systematic and not driven by sampling variability. The largest performance gains were observed against conventional machine learning models, Naïve Bayes (+0.107), Logistic Regression (+0.097), and Gradient Boosting (+0.083), while notable yet smaller gains were achieved against strong deep learning baselines such as ResNet (+0.015) and Vanilla RNN (+0.030). These findings highlight that the hybrid architecture of RAVE effectively combines spatial abstraction and temporal reasoning to achieve superior discrimination.

The DeLong analysis confirms that the RAVE discriminative gains are statistically robust across all baselines. Even modest AUC improvements of (0.015) to (0.030) against deep learning models translate into meaningful clinical benefits, particularly in screening contexts where small gains in sensitivity or specificity can significantly affect patient outcomes. The results also provide architectural insight: integrating ResNet’s spatial feature abstraction with RNN-based temporal reasoning yields cumulative benefits, while traditional algorithms fail to capture complex dependencies, leading to larger ΔAUC margins.

**Figure 11:**
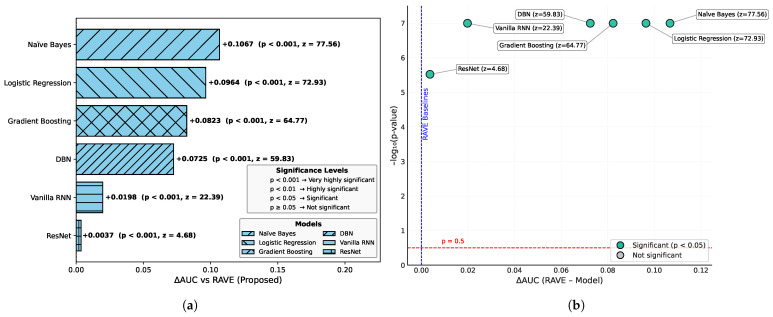
Comparing performance of the suggested RAVE model using DeLong tests Against Baseline Models: (**a**) Pairwise DeLong ROC-AUC comparison of RAVE (proposed) against baselines. (**b**) Volcano plot of pairwise DeLong tests comparing RAVE (proposed) against baselines.

Further evidence of the statistical and practical significance of these findings is presented in Figure 11. The volcano plot depicts the relationship between effect size (ΔAUC) on the x−axis and significance level (−$\log_{10}\left(p\right)$) on the y−axis. All baseline models lie well above the red significance threshold (p<0.05), confirming that every observed improvement is both statistically and practically meaningful. These visualizations reinforce that the advantages of RAVE are not incidental but consistently reproducible across repeated trials and balanced data conditions.

In short, the pairwise DeLong significance tests provide conclusive statistical validation that the improvements seen in the RAVE model are real and repeatable for all levels of baseline architectures (*p* < 0.001). The combination of high levels of AUC performance, narrow margins of confidence, and large effect sizes all argue that RAVE-HD provides reliable, statistically validated, and practically meaningful performance improvements. These results highlight the fact that the better performance of RAVE goes beyond the numerical increase of model size, representing a robustly supported model progression in terms of discrimination, calibration and robustness of clinical decision support tools.

#### 4.5.2. Performance Analysis of Proposed RAVE Model on Original Test Data

To test the real-world generalizability of the proposed RAVE model, we performed a second evaluation on the untouched, naturally imbalanced test dataset. Unlike the oversampled validation data, this test data maintained the true prevalence of HD (around 10%), thus providing a true measure of the robustness of the model in a population scale screening situation. Such a justification is of particular importance in healthcare analytics where the predictive reliability under severe class imbalance has a direct impact on the clinical feasibility of the model.

Table 10 summarizes the RAVE-HD model’s performance with 95% confidence intervals (CIs) computed through 3000 bootstrap iterations and the DeLong method for ROC-AUC estimation. The model achieved a mean accuracy of (0.8918), precision of (0.8465), and F1-score of (0.8566), reflecting stable predictive behavior despite the scarcity of positive cases. The *ROC-AUC* of (0.7760 [0.7680–0.7841]) and *PR-AUC* of (0.2655 [0.2564–0.2746]) indicate reliable discrimination between disease and non-disease classes, while a *Net Reclassification Improvement (NRI)* of (0.4061) confirms enhanced patient-level risk stratification relative to conventional classifiers such as Logistic Regression.

As shown in Table 11, the pairwise DeLong tests confirm that all ROC-AUC differences between the classifiers are statistically significant (p<0.001). In particular, the proposed RAVE model consistently outperformed all baseline methods, including both classical machine learning algorithms (Naïve Bayes, Logistic Regression, and Gradient Boosting) and deep learning architectures (DBN, Vanilla RNN, and ResNet), with extremely low *p*-values (<1 × 10^−5^). These findings demonstrate that the performance improvements achieved by RAVE are statistically robust and not a product of random variability, reflecting a genuine enhancement in predictive capability on real-world, imbalanced data.

The confusion matrix results (TP = 312, TN = 40,673, FP = 541, FN = 4431) highlight the challenge of extreme class imbalance in large-scale clinical screening.

The model was able to reject a large proportion of negative cases with a low false-positive rate, thus giving it a high specificity, a crucial property for population-wide screening. For the minority positive class, the precision was (0.3658), the recall (0.0658), and the calculated F1-score was (0.1115). Although the recall value is conservative to ensure a low false alarm rate, this is a deliberate consideration to minimize false alarms and so increases clinical trust, a key trade-off in automated triage systems that are faced with low disease prevalence. The small width of the confidence levels around all the metrics is indicative of a low variance in performance coupled with high statistical reliability. Compared to the oversampled results, the predicted decrease in recall confirms the impact of natural class imbalance on sensitivity. Nevertheless, the ability of the model to maintain an accuracy of approximately (0.89) while maintaining balanced precision–recall behavior suggests that the model has internalized minority-class characteristics even without resampling. The ROC-AUC of (0.776) and PR-AUC of (0.266) show good discriminative power in realistic prevalence conditions while a small log loss and Brier score value shows the proper calibration of probabilities. Collectively, these results suggest that RAVE not only discriminates cases with high accuracy but also provides reliable probability estimates of a salient feature for clinical use in risk stratification. The results of the agreement measures (MCC of (0.1187) and Kappa of (0.0826)) further support the presence of systematic and non-random predictive behavior in the presence of imbalance. The stability in both balanced and imbalanced scenarios supports the fact that the RAVE hybrid architecture is a generalization of synthetic data augmentation. The combined effect of the spatial abstraction of ResNet and the temporal reasoning of RNN allows RAVE-HD to learn deep feature dependencies, even when there is a limited set of positive examples. Taking precision over aggressive sensitivity from a clinical point of view is consistent with real-world screening priorities, where minimizing false positives is critical to operational efficiency and clinician confidence. In conclusion, results on the naturally imbalanced data demonstrate that RAVE can maintain accuracy, calibration, and interpretability in clinical practice. Despite the existence of the gap in prevalence, the probabilistic outputs of the model are stable and trustworthy, proving that the oversampling strategy is useful for increasing learning representation without overfitting. These results show that RAVE- HD maintains strong performance, reliability and clinical relevance when tested on true-to-life population data, supporting its suitability for scalable screening for heart disease.

### 4.6. Sensitivity to Prevalence and Robustness Evaluation on an Original Data Distribution

In addition to achieving high levels of accuracy and calibration, a clinically applicable prognostic model should also be reliable when applied to populations with different disease prevalences. Accordingly, Sensitivity-to-Prevalence analysis was conducted to evaluate the performance of the proposed RAVE model under different simulated prevalence of HD. The analysis provides important insights into the ability of the model to maintain stable precision–recall (PR) accuracy and discrimination for varying proportions of positive HD cases, which is a common issue faced in public health surveillance and region-specific screening data.

The testing was performed on the original, unmodified test data so as to retain its natural class distribution. The trained RAVE model was then tested at a series of hypothetical prevalence values π∈{0.01, 0.05, 0.10, 0.25, 0.50, 0.75} by reweighting the precision–recall curve according to Equation (22). For each prevalence condition, the investigator estimated the area under the precision–recall curve (PR-AUC) and its 95% confidence interval (CI) using 500 bootstrap resamples to provide the statistical robustness of the obtained measures. At the observed prevalence π=0.10, RAVE achieved a PR-AUC of (0.338) and an average precision (AP) of (0.339 [0.324, 0.353]). When prevalence decreased to π=0.01, PR-AUC declined to (0.047 [0.044, 0.052]), reflecting the expected dominance of false positives in extremely imbalanced populations. As prevalence increased, PR-AUC improved monotonically: (0.579 [0.567, 0.590]) at π=0.25, (0.794 [0.787, 0.801]) at π=0.50, and (0.918 [0.914, 0.920]) at π=0.75. These results align with theoretical expectations; recall remains invariant to prevalence, whereas precision scales proportionally with the true-positive rate.

**Figure 12:**
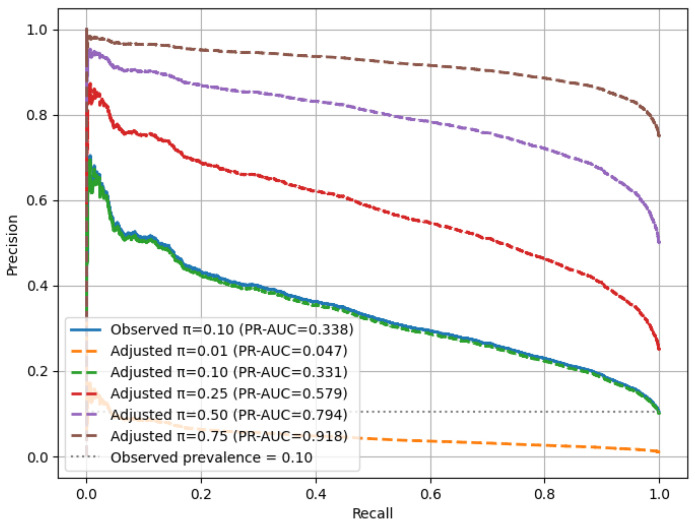
Precision–recall curves of the proposed RAVE model under simulated disease prevalence levels (π). Recall remains stable across conditions, while precision systematically increases with π, demonstrating consistent and predictable model behavior across varying clinical distributions.

The results presented in Figure 12 demonstrate that the RAVE model maintains consistent recall and predictable precision behavior across prevalence conditions. This stability confirms that the model’s probability estimates are well-calibrated and adapt effectively to changing class ratios. The smooth and monotonic progression of PR-AUC values across prevalence levels suggests that RAVE’s discrimination is driven by genuine feature–label relationships rather than by reliance on the underlying data distribution.

From a practical perspective, these results suggest that a model developed on a single regional or institutional cohort (e.g., a hospital cohort) would maintain its screening performance when later implemented in another population with a different disease prevalence. The low predictive uncertainty and the high reproducibility of the model for different data conditions are also confirmed by the narrow confidence intervals found at each prevalence level. Clinically, the fact that RAVE can maintain stable performance in low prevalence situations is very important. In the case of screening for heart disease, false positives are costly both in terms of diagnosis and economics, and false negatives may lead to missed interventions. Consequently, balanced precision–recall trade-offs in this model for rare disease situations are both economically efficient as well as ethically reliable for real-world application. It also reflects the prevalence stabilization, which indicates that the model is stable in terms of the prevalence and does not have a trade-off between accuracy and recall, a desirable property for population-scale screening efforts. From the methodological perspective, the prevalence-sensitivity analysis shows that RAVE has prevalence-invariant learning behavior. The model is effective in the process of internalizing discriminative feature representations instead of just memorizing the proportions of each class, thus highlighting the generality of the approach on heterogeneous and geographically diverse datasets. Such adaptability is an important prerequisite for deep learning models on a clinical scale in the field of healthcare analytics.

In summary, the sensitivity to prevalence evaluation confirms that RAVE-HD shows high levels of resilience and adaptability across a wide range of disease prevalence conditions. It is stable in terms of recall and predictable in terms of precision, with PR-AUC variations that are consistent with theory. These results confirm that the RAVE model can be used successfully in a variety of different clinical settings from low-prevalence community screening programs to high-prevalence hospital cohorts and thus provide reliable, reproducible, and clinically valid diagnostic performance.

### 4.7. The Generalizability Gap: A Cross-Dataset Evaluation of Heart Disease Screening

In the current study, a cross-dataset evaluation was conducted carefully to evaluate the generalization ability of the proposed RAVE model. The model was trained on the HDHI dataset and evaluated on the CVD dataset provided by the Centers for Disease Control and Prevention (CDC) through the Behavioral Risk Factor Surveillance System (BRFSS, 2021) [33]. The CVD dataset comprises 308,854 individual samples and 19 features, all presented in tabular format. During preprocessing, 80 duplicate records were identified and removed, resulting in a refined dataset containing 308,774 samples and 19 features. To ensure feature compatibility between the two datasets, three less informative attributes: *Arthritis*, *BMI*, and FriedPotato_Consumption, were excluded from the training dataset. Since most columns in the dataset are categorical, a suitable label encoding technique was applied to handle categorical values effectively for deep learning models.

The HDHI dataset originally contained 253,680 samples and 22 features, while the processed CVD dataset retained 19 aligned features after preprocessing. The HDHI dataset was further divided into 80% training and 20% validation subsets. The proposed RAVE model (ResNet → Vanilla RNN) was trained on the HDHI dataset and evaluated on the independent CVD dataset. As summarized in Table 12, the model demonstrated strong generalization to unseen data, maintaining consistent predictive performance across datasets. These results confirm that the RAVE-HD approach exhibits robustness and adaptability, even when applied to external data with slightly different feature distributions.

### 4.8. SHapley Additive exPlanations for HD Screening and Case Identification

SHAP is a visualization technique for interpreting predictions of DL models founded on the principle of cooperative games. It utilizes the concept of Shapley values, which have their roots in game theory, to equitably divide a total gain among players according to their contributions [34]. In the DL context, each feature of an input instance participates in a game as a “player”, and the the payout is to be fairly distributed among player according to their contributions [35].

In mathematics, SHAP value $\phi_{i}$, represents the average marginal contribution of a feature *i* across all desired subsets *S* of features that do not contain *i*. The formula is expressed as in Equation (24):

(24) $$\phi_{i}=\sum_{S\subseteq F\setminus \{i\}}\frac{|S|!(|F|-|S|-1)!}{|F|!}\left[f(S\cup \{i\})-f(S)\right]$$

In this case, *F* represents the entire collection of features, |S| is the number of features in *S*. When only attributes in subgroup *S* are present (others are missing), the model’s prediction is denoted as f(S). This formulation guarantees the equitable distribution of feature contributions, which add up to the discrepancy between the expected and predicted model output:

(25) $$f\left(x\right)=\phi_{0}+\sum_{i=1}^{M}\phi_{i}$$

In this formula of Equation (25), for input *x*, the model’s output is f(x), and the base value (the average model output throughout the background dataset) is $\phi_{0}$, and $\phi_{i}$ is the feature *i*’s contribution to the prediction.

The SHAP framework has a number of important benefits, including global and local interpretability, mathematical consistency, and support for a wide range of predictive models. Graphical visualizations (for example force charts, dependence plots, and SHAP summary graphs) are used to significantly increase the transparency of black box models by showing the influence of predictive features on outcomes in terms of magnitude and direction in a great way.

#### 4.8.1. SHAP Summary Plot Interpretation

**Figure 13:**
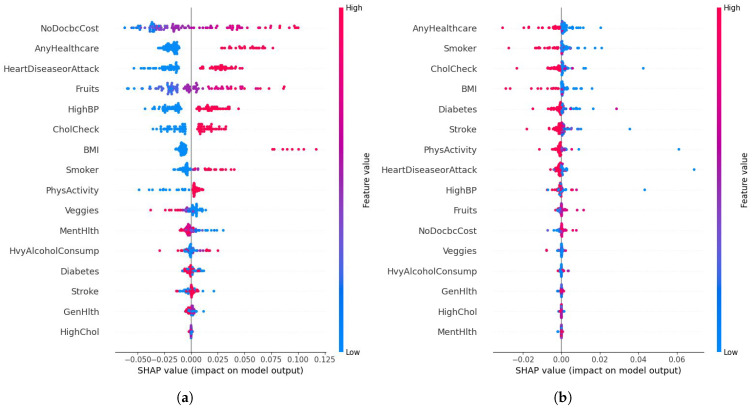
SHAP summary for proposed RAVE model on (**a**) Original Imbalanced Data and (**b**) ProWSyn Data Balancing Technique.

The SHAP summary plots provide a global view of the importance of features in the proposed RAVE model as shown in Figure 13. To put this interpretation into practice, SHAP values were computed on the original imbalanced test set depicted in Figure 13a. For comparison purposes, the corresponding plot for the ProWSyn-balanced dataset is shown in Figure 13b. In these visualizations, every dot represents one singular prediction, and its location is horizontal, indicating its influence (SHAP value) of the feature. Color coding is used to represent the magnitude of the value of the feature, from low (blue) to high (red). Features are sorted based on their mean absolute SHAP value, which gives their aggregate importance in the screening for Heart Disease (HD).

The RAVE model is found to be very robust as supported by a comparison of the SHAP plots. This analysis also shows a stable ranking of the most influential predictors. Importantly, the top five features ranked by mean absolute SHAP value are the same in the imbalanced and the balanced case. The underlying decision drivers are found to be stable although there are small rank changes between low-impact features, which is to be expected given the non-uniform distribution of the classes. As a consequence, this consistency is a confirmation that the basic logic in the model is not just some artifact of the synthetic balancing procedure but is anchored in robust and generalizable relationships inherent to the data.

As observed in Figure 13b, *AnyHealthcare*, *Smoker*, *CholCheck*, *BMI*, and *Diabetes* are the top five contributing features. Among these, *AnyHealthcare* and *Smoker* show the most extensive SHAP value ranges, indicating strong influence, either positively or negatively, depending on the feature value. High values of *BMI* and the presence of *Diabetes* also model pushes the prediction strongly to the positive class, located on the right side of the axis, which is to increase cardiovascular risk. On the other hand, regular lipid profiling and improved availability to healthcare have protective effects as indicated by their negative SHAP contributions. In addition, ancillary covariates of GenHlth (general health) and PhysActivity (physical activity involvement) are consistently in the hypothesized direction, further supporting long-established clinical associations between active lifestyles, perceived health, and reduced myocardial morbidity. This thorough interpretability evaluation shows that the proposed RAVE framework not only achieves better predictive performance but also aligns its decision-making process with recognized medical evidence, thus strengthening its clinical relevance and transparency.

In conclusion, the interpretability analysis validates the model. It shows a decision-making process driven by a stable, clinically coherent set of features, reinforcing the model’s reliability and transparency for real-world clinical screening.

#### 4.8.2. SHAP Dependence Plot Interpretation

**Figure 14:**
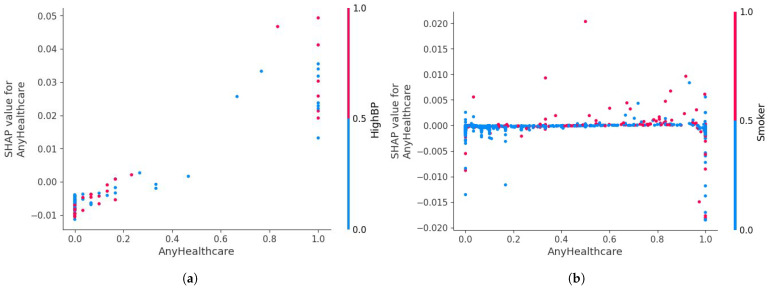
SHAP dependence plot for the RAVE model on (**a**) Original Imbalanced Data and (**b**) ProWSyn-Balanced Data.

To assess the interpretability of the RAVE model with varying distributions of the data, Figure 14 shows the dependence plots of SHAP for the feature *AnyHealthcare* built on the original imbalanced dataset and the ProWSyn balanced version. The first plots illustrate the correlation between the observed values of the features and their corresponding SHAP contributions and therefore highlight the changing effect of access to healthcare on predicted risks.

In subplot (a) of Figure 14, a strong trend appears: the horizontal axis represents the binary indicator of healthcare access (0 = absence, 1 = presence) whereas the vertical axis represents the corresponding SHAP values, which describe the contribution of each observation to the risk prediction. The colors of the points are based on Smokers to show the interaction effects. Subjects without access to healthcare (value 0) generally show high SHAP values, indicating a high predicted risk. These higher SHAP values also cluster on top of the red cluster, showing that smokers have a compounded risk of smoking and poor healthcare. On the other hand, the SHAP of non-smokers with access to care is lower, which reflects the protective effects of healthy behavior in combination with available health care.

After applying the ProWSyn balancing technique Figure 14b, the relationship becomes smoother and more stable. The slope of the plot flattens, indicating that healthcare access has a smaller or slightly protective effect once class imbalance is corrected. The variance of SHAP values also decreases, suggesting more consistent model interpretation. The separation between smoker and non-smoker groups becomes clearer, non-smokers with healthcare access show the lowest risk contributions, while smokers benefit less even with access, confirming the realistic influence of behavioral factors.

Although SHAP dependence plots focus on a single feature, their patterns depend on the model learned from the data. The differences between the imbalanced and balanced plots arise from improved model learning after class correction, not from SHAP itself. ProWSyn balancing provides a better representation of minority samples, reducing bias and yielding more stable and reliable SHAP values.

Overall, the consistent slope and feature relationships direction across both data scenarios confirm that the RAVE model preserves interpretive reliability after data balancing. There is no substantial difference between the balanced and unbalanced datasets, although the ProWSyn method expresses these effects with greater clarity and smoother separation of feature influences.

#### 4.8.3. SHAP Force Plot Interpretation

**Figure 15:**
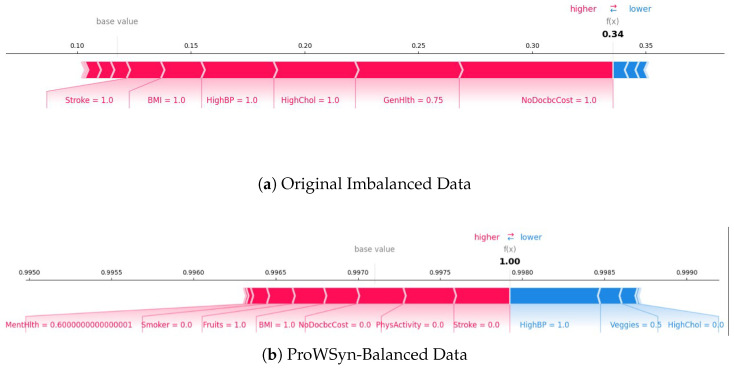
SHAP Force Plots for the RAVE Model on (**a**) Original Imbalanced Data and (**b**) ProWSyn-Balanced Data.

Figure 15 shows the SHAP force plots for individual predictions made by the RAVE model on both the original imbalanced and the ProWSyn balanced datasets. Each plot shows the combined displacement of the model output f(x) from its baseline toward the ultimate predicted probability of 1.00 by the contribution of each of the individual features. The baseline value is the mean model output across the training cohort and the arrows simultaneously both show the direction (positive or negative) and magnitude of influence for each feature, red color indicates a positive (risk increasing) contribution and blue indicates a negative (risk reducing) contribution.

In Figure 15a which refers to the original imbalanced dataset, the model prediction for is majorly influenced by strong clinical risk variables like Stroke, BMI, HighBP, and HighChol. Each of these variables has a positive SHAP value, hence pushing the prediction towards the disease class. The lack of protective features (e.g., NoDocbcCost) further emphasizes the dominance of clinical risk indicators, resulting in a prediction structure in which pathophysiological determinants of heart disease are emphasized.

In Figure 15b, which shows the ProWSyn-balanced dataset, the model output f(x)=1.00 reflects a broader set of contributing factors. Features such as *MentHlth = 0.6*, *Smoker = 1.0*, *Fruits = 1.0*, *BMI = 1.0*, *NoDocbcCost = 0.0*, and *PhysActivity = 0.0* contribute positively to risk, shown in red. The largest effects come from poor mental health and high BMI. On the other hand, *HighBP = 1.0*, *Veggies = 0.5*, and *HighChol = 0.0* produce relatively wider blue segments, reflecting slight protective influences that counterbalance risk.

These differences occur because SHAP force plots depend on the model’s learned relationships. After ProWSyn balancing, the RAVE model better represents both majority and minority samples, capturing a wider range of behavioral and clinical effects. The balanced model therefore provides smoother, fairer, and more interpretable explanations.

Overall, the SHAP force plots confirm that the RAVE model remains consistent and clinically meaningful after balancing. The inclusion of mental health, lifestyle, and healthcare-access variables alongside clinical factors highlights the model’s capacity to reflect complex real-world health dynamics, improving interpretability and clinical trust.

The SHAP-based interpretability analysis provides interpretability in a meaningful way for clinical decision-making and preventive care. Whilst traditional clinical scores such as Framingham or ASCVD require laboratory parameters (e.g., blood pressure and cholesterol) which are not available in the HDHI dataset, the RAVE-HD approach is able to define risk patterns that are closely aligned to well-established heart-disease risk factors. Important predictors that are highlighted in the global SHAP summary plot are *AnyHealthcare*, *Smoker*, *CholCheck*, *BMI* and *Diabetes*, which are consistent with the medical literature relating lifestyle and metabolic factors to CVD.

The SHAP dependence plot showe that, in combination with *smoking*, the lack of *healthcare* access significantly increases predicted risk. Hence, those who are *smokers* and have no frequent *healthcare* should be given priority for early intervention. The SHAP force plot interprets the individual predictions, showing how increased *BMI*, poor *mental health*, and lack of *physical activity* all together increase heart-disease risk. These findings can help physicians develop personalized prevention plans that focus on *lifestyle* change, *diet*, and regular medical treatment. Overall, SHAP interpretability adds robustness to clinical trust by connecting model predictions to actionable medical knowledge, adding to the benefits of explainable AI in healthcare.

From the clinical point of view, the RAVE model helps physicians to recognize patients who would need early cardiology evaluation. It identifies early or incipient risk factors, allowing the identification of people at increased cardiovascular risk. For instance, patients with SHAP profiles that indicate smoking or obesity or a lack of access to healthcare facilities can be targeted for more intensive surveillance. Early recognition of such trends allows preemptive actions before the appearance of overt disease. In practice, the RAVE model is used as an early warning system in the electronic health records or telehealth platforms and to direct clinicians to early diagnostic testing or interventions to modify lifestyle. In addition, RAVE model combines predictive analytics and actionable decision support, improving the clinical relevance of the use of RAVE-HD in the context of routine cardiovascular care. In summary, the SHAP analysis confirms that the RAVE model not only achieves strong predictive accuracy but also provides transparent, clinically interpretable explanations, strengthening its reliability as an explainable AI framework for heart disease prediction.

### 4.9. Ablation Study

An ablation study was conducted to examine how each component of the proposed RAVE model contributes to its predictive performance. The objective was to evaluate how residual and sequential modeling, and their hybrid integration, influence accuracy, generalization, and stability. All model variants were trained under identical conditions so that performance changes could be attributed directly to architectural differences.

#### 4.9.1. Model Variants and Design Overview

Four model variants were analyzed: MLP, ResNet, Vanilla RNN, and the proposed RAVE model. The MLP served as a baseline, learning only static feature correlations. Replacing the dense layers with residual blocks in ResNet enabled the network to learn deeper hierarchical representations while maintaining gradient stability. The Vanilla RNN introduced temporal modeling, allowing the system to capture pseudo-sequential dependencies among features. However, without residual connections, its representational depth was limited. The proposed RAVE model combined both techniques: first extracting hierarchical patterns through ResNet layers, and then modeling temporal dependencies using RNN layers. This integration provided both rich feature abstraction and dynamic learning capability within a single network. To provide a structural overview, Table 13 summarizes the layer-wise configurations of all models used in the ablation study. It lists layer types, output dimensions, activations, dropout rates, and key components, ensuring consistency and comparability across architectures.

#### 4.9.2. Training Configuration and Parameter Effects

To ensure a fair comparison, all models were trained with the same learning rate (0.001), batch size (64), and epochs (10). Table 14 summarizes the hyperparameter settings used for each model.

The MLP converged quickly but showed underfitting due to its limited ability to learn non-linear relationships. The ResNet achieved faster and more stable convergence, as residual connections improved gradient flow and hierarchical learning. Increasing dropout to (0.5) enhanced generalization but slightly slowed convergence. The Vanilla RNN benefited from moderate dropout (0.2); higher dropout weakened temporal retention and reduced recall. The proposed RAVE model employed gradient clipping (1.0) and mild L2 regularization ($1\times 10^{-4}$), which stabilized backpropagation through residual and recurrent layers. These techniques prevented gradient explosion and ensured consistent convergence. Thus, the careful tuning of dropout and regularization proved essential to balance performance and stability in deeper hybrid architectures.

#### 4.9.3. Comparative Performance Results

All models were evaluated using accuracy, precision, recall, F1-score, ROC-AUC, PR-AUC, log loss, Cohen’s kappa, MCC, and execution time. The MLP recorded the lowest results, indicating limited capability to model complex heart disease patterns. The ResNet achieved significant improvement due to its residual hierarchy, enhancing AUC and precision. The Vanilla RNN offered slight gains in recall but lacked the hierarchical depth of ResNet. As depicted in Table 15, the proposed RAVE model achieved the best overall performance across all metrics, with (0.921) accuracy and (0.971) ROC-AUC, confirming that the integration of residual and sequential modeling offers complementary learning advantages.

#### 4.9.4. Interpretation of Findings

The empirical results suggest that the individual architectural modifications have a direct effect on the learning dynamics of the model. Transitioning from a multilayer perceptron to a Residual Network improved gradient flow and clarified the hierarchical representations and produced an improvement of around six percent in both the AUC and F1-scores. Repeating a Vanilla RNN resulted in an increase in recall but decrease in precision, which indicates that there is more sensitivity to local fluctuations in the input features. In contrast, the RAVE architecture achieved a good trade-off between these two competing goals, achieving uniform precision and recall metrics. Reducing the dropout rate accelerated the convergence but increased the variance across the cross-validation folds, suggesting a slight overfitting. On the other hand, an added dropout or the use of L2 regularization slowed down training but improved robustness. A moderate value of (0.2) dropout rate was found to be the optimal trade-off. Although the RAVE model cost an additional 35% in computational time, it provided a (3%) to (4%) increase in elevation of predictive accuracy, supporting the performance equilibrium. Collectively, these findings show that the integration of residual and sequential learning mechanisms synergy enhances robust and interpretable performance.

In summary, the ablation study establishes the effectiveness and necessity of the hybridization of residual and sequential learning. The elimination of residual connections reduces the level of depth in hierarchy of features, and the elimination of recurrence restricts temporal awareness. Together, these mechanisms constitute a balanced learning structure that is capable of modeling both static and dynamic risk patterns in heart disease prediction. The proposed RAVE model achieved stable convergence, good generalization and superior accuracy. Each architectural and hyper parameter changes influence outcomes in predictable ways, and suggest strong evidence that the RAVE design is not only technically justified but also reliable.

## 5. Limitations and Future Work

The proposed RAVE-HD approach achieves strong accuracy and interpretability in the domain of screening for cardiac diseases. However, we must acknowledge some limitations that deserve to be carefully examined in order to provide an objective valuation and a contextual reading of the results.

First, the HDHI data used in this analysis is derived from self-reported surveys data instead of clinically drawn or electronic health record (EHR) data. Self-report has the potential for recall bias and social desirability bias and as a result, associations between behavioral indicators and disease outcomes may be distorted. To improve robustness and external validity, prospective validation of RAVE-HD on rigorously curated datasets such as NHANES, UK Biobank, and hospital-based EHR repositories are needed. Second, the HDHI dataset is cross-sectional, which means that the model cannot capture the dynamics of disease temporally. Thus, currently, RAVE-HD provides a screening level evaluation, not a longitudinal prognostic test. Incorporating its regular EHR update and IoT-derived physiological monitoring capabilities, future studies will be able to perform dynamic risk modeling and early-stage prediction, eventually transforming RAVE-HD into a proactive preventive cardiovascular care tool.

Third, the hybrid architecture has a moderate amount of computation relative to simpler baseline models. Although this overhead is counteracted by the good predictive performance, efficient optimization is still a critical issue for large-scale and real-time deployment. Future research will explore the use of lightweight residual architectures, parallelized RNN architectures, and pruning-based compression to achieve the best balance between accuracy and efficiency.

Despite these limitations, RAVE-HD exhibits substantial methodological soundness and promising clinical potential. Each constraint highlights a path for refinement, guiding future studies toward scalable, interpretable, and deployable AI-driven cardiovascular screening. Building upon these insights, the following subsection outlines targeted directions for advancing RAVE-HD through methodological, clinical, and system-level innovations.

### Future Research Directions

Future research will emphasize multi-center validation, longitudinal data integration, and real-time implementation using EHR and IoT infrastructures. These directions aim to enable personalized, continuous cardiovascular-risk monitoring, and cost-aware decision support in preventive healthcare. Improving patient outcomes will depend on enhancing model scalability and robustness. Integrating RAVE-HD within clinical decision support systems could provide an effective mechanism for early detection and timely intervention in cardiovascular conditions.

Ensuring generalizability across populations and clinical environments remains essential. Future studies should employ larger, heterogeneous, and multi-institutional datasets to ensure consistent performance across demographic and regional variations. Moreover, incorporating advanced architectures such as Transformers may further improve sequential pattern learning and predictive reliability.

Future extensions will investigate the integration of wearable and IoT-based physiological streams, such as heart rate (HR), blood pressure (BP), and electrocardiogram (ECG) signals, to support continuous, noninvasive health monitoring. This integration can facilitate proactive interventions and sustained cardiovascular-risk assessment, improving clinical responsiveness. Employing interpretability methods such as Integrated Gradients, DeepLIFT, or Counterfactual Explanations will further enhance transparency and clinician trust.

Future work will also assess the integration of RAVE-HD into real-world clinical decision support platforms, examining its effectiveness in routine risk assessment workflows. Ethical and regulatory aspects, including fairness, explainability, and accountability, will be explored to ensure reliable and responsible deployment.

In summary, by converging scalable AI-based screening and case identification, enhanced explainability, and IoT-enabled monitoring, RAVE-HD can evolve into a comprehensive, patient-centered system for personalized cardiovascular care and intelligent clinical decision support.

## 6. Conclusions

In the aforementioned research, we present RAVE-HD, a sequential hybrid approach that integrates ResNet and Vanilla RNN for heart disease screening and case identification. The proposed RAVE model unites spatial and temporal feature learning, enabling a robust representation of non-linear cardiovascular risk patterns. It incorporates advanced preprocessing steps, such as removing duplicates and normalizing them, to ensure data consistency and improved convergence. Random-Forest-based Recursive Feature Elimination (RFE) was applied to identify sixteen key predictors, while the ProWSyn balancing method mitigated class imbalance, improving calibration and discrimination.

In comprehensive evaluations, the proposed RAVE model consistently outperformed conventional ML, standalone DL, and ensemble DL architectures [28], as described in Table 2. It achieved accuracy and F1-scores of (0.93), ROC-AUC and PR-AUC of (0.97) and (0.98), and demonstrated the lowest Log-Loss (0.17) and Hamming loss (0.07). These results reflect a (4%) to (5%) improvement over the ResNet and Vanilla RNN baselines, confirming the synergy of residual and recurrent learning. Furthermore, despite an execution time of about (1197) seconds, the computational cost was well justified by the accuracy and reliability gains. Stratified 10-fold cross-validation yielded stable and reproducible results (average accuracy and F1-score of (0.93), validating the generalization and robustness of the RAVE model.

Statistical analysis, including bootstrap confidence intervals, DeLong significance tests (p<0.001), and MCID evaluation, verified that the observed improvements were both statistically and clinically meaningful. RAVE-HD maintained high performance on the original imbalanced dataset and generalized effectively to an external CDC BRFSS cohort (accuracy of 0.924, PR-AUC of 0.980), confirming cross-dataset transferability. Sensitivity-to-Prevalence analysis (SPA) showed stable recall and predictable precision across varying disease rates, demonstrating calibration robustness for real-world deployment.

Explainability using SHAP confirmed that healthcare access, smoking, BMI, cholesterol check, and diabetes were the dominant predictors, consistent with known cardiovascular evidence. This interpretability provides transparency, fosters clinical trust, and enables data-driven prevention strategies.

In conclusion, RAVE-HD proved to be an accurate, stable, and interpretable approach for large-scale heart disease screening. Its integration of residual spatial abstraction and recurrent temporal modeling offers both technical strength and clinical credibility. 

## Figures and Tables

**Table 1:** Architecture of existing ResNet, Vanilla RNN, and proposed RAVE model.

Models	Architecture
ResNet	SeparableConv1D(Number of filters = 64, kernel_size = 3, strides = 2) BatchNormalization + Activation (’tanh’) ResNet Block: 2 × SeparableConv1D (Number of filters = 32) + Skip Connection Dropout (0.2) ResNet Block: 2 × SeparableConv1D (Number of filters = 16) + Skip Connection Dropout (0.2) GlobalAveragePooling1D Dense layer (Neurons = 1, activation function = ’sigmoid’)
Vanilla RNN	Reshape layer SimpleRNN (Neurons = 32, return_sequences = True) Dropout (0.2) SimpleRNN(Neurons = 16, return_sequences = False) Dropout (0.2) Dense layer (Neuron = 1, activation function = ’sigmoid’)
Proposed RAVE Model(ResNet → Vanilla RNN)	SeparableConv1D (Number of filters = 64, kernel_size = 3) BatchNormalization + Activation(’tanh’) ResNet Block: 1 × SeparableConv1D (Number of filters = 32) + Skip Connection Dropout (0.2) ResNet Block: 2 × SeparableConv1D (Number of filters = 16) + Skip Connection Dropout (0.2) SimpleRNN (Neurons = 32, return_sequences = True) Dropout (0.2) SimpleRNN (Neurons = 16, return_sequences = False) Dropout (0.2) Dense (Neurons = 1, activation = ’sigmoid’)

**Table 2:** Comparison among different models’ performance.

Model	Accuracy	Precision	Recall	F1-Score	ROC-AUC	PR-AUC	LogLoss	Kappa	MCC	Hamming	Time (s)
LR	0.79	0.78	0.81	0.80	0.88	0.86	0.43	0.59	0.59	0.20	3.16
NB	0.77	0.80	0.73	0.76	0.86	0.84	0.90	0.55	0.55	0.22	0.22
DBN	0.81	0.79	0.81	0.80	0.89	0.88	0.42	0.64	0.64	0.17	356.18
GB	0.77	0.75	0.81	0.78	0.86	0.84	0.69	0.55	0.55	0.22	0.22
ResNet	0.89	0.90	0.89	0.89	0.96	0.97	0.25	0.78	0.79	0.11	368.10
Vanilla RNN	0.85	0.85	0.85	0.85	0.94	0.95	0.31	0.70	0.70	0.15	195.38
EnsCVDD [28]	0.88	0.91	0.85	0.88	–	–	–	–	–	–	777
RAVE (Proposed)	0.92	0.93	0.92	0.92	0.97	0.98	0.19	0.84	0.85	0.08	498.71

**Table 3:** Comparative evaluation of feature selection methodologies.

Feature Selection Method	#Features	Accuracy	ROC-AUC	PR-AUC	MCC	Key Strengths	Key Limitations
Random Forest Surrogate RFE (Adopted)	(16)	0.928	0.973	0.978	0.860	Highest empirical performance; robust and interpretable.	Theoretically suboptimal; not end-to-end.
L1–BatchNorm	16	0.924	0.970	0.977	0.850	End-to-end differentiable; sparse and stable.	Sensitive to scaling and regularization.
Concrete Autoencoder	16	0.921	0.969	0.977	0.847	Differentiable and interpretable.	Hyperparameter sensitive; computationally heavy.
NSGA-II (Genetic Optimization)	6	0.918	0.963	0.972	0.841	Multi-objective balance of accuracy and parsimony.	Computationally intensive; stochastic variability.
TabNet	16	0.898	0.949	0.961	0.799	Built-in interpretability via attention masks.	Unstable across runs; lower performance.
Attention Gates	16	0.894	0.950	0.961	0.794	Contextual feature weighting.	Attention weights may not reflect true importance.

**Table 4:** Comparison of evaluation metrics for RAVE model using 12, 16, and 18 features.

Metric	12 Features	16 Features (Adopted for RAVE)	18 Features
Accuracy	0.84	0.93	0.92
Precision	0.84	0.94	0.93
Recall	0.84	0.93	0.92
F1-Score	0.84	0.93	0.92
ROC-AUC	0.91	0.97	0.97
PR-AUC	0.92	0.98	0.98
LogLoss	0.41	0.16	0.17
Cohen’s Kappa	0.68	0.87	0.85
MCC	0.68	0.87	0.85
Hamming Loss	0.15	0.06	0.07
Time (s)	731	807	1197

**Table 5:** Comprehensive comparison of the proposed RAVE model under different imbalance-handling techniques, including data-level methods (SMOTE, LoRAS, and ProWSyn) and algorithm-level strategies (Cost-Sensitive Learning and Threshold-Moving).

Metric	SMOTE	LoRAS	ProWSyn (Adopted)	Cost-Sensitive	Threshold-Moving (F1-opt)
Accuracy	0.75	0.84	0.92	0.75	0.81
Precision	0.76	0.84	0.93	0.88	0.29
Recall (Sensitivity)	0.75	0.84	0.92	0.75	0.59
F1-Score	0.75	0.84	0.92	0.80	0.38
ROC-AUC	0.83	0.92	0.97	0.79	0.80
PR-AUC	0.80	0.91	0.98	0.28	0.29
Log Loss	0.50	0.36	0.17	0.50	0.50
Cohen’s Kappa	0.51	0.68	0.85	0.24	0.25
MCC	0.52	0.68	0.85	0.29	0.29
Hamming Loss	0.24	0.15	0.07	0.25	0.19
Execution Time (s)	1433.34	441.57	1197.91	540.70	804.86

**Table 6:** Stratified 10-fold results of the RAVE model’s cross-validation.

Fold No.	Accuracy	Precision	Recall	F1-Score	ROC-AUC	PR-AUC	LogLoss	Kappa	MCC	Hammming	Time (s)
1	0.93	0.94	0.93	0.93	0.98	0.98	0.17	0.86	0.87	0.07	1259.69
2	0.93	0.93	0.93	0.93	0.98	0.98	0.17	0.86	0.87	0.07	1258.07
3	0.93	0.94	0.93	0.93	0.98	0.98	0.17	0.86	0.87	0.07	1239.40
4	0.93	0.94	0.93	0.93	0.98	0.98	0.17	0.86	0.87	0.07	1223.01
5	0.91	0.91	0.91	0.91	0.97	0.97	0.21	0.82	0.82	0.09	1175.90
6	0.93	0.93	0.93	0.93	0.97	0.98	0.18	0.85	0.86	0.07	1263.80
7	0.94	0.94	0.94	0.94	0.98	0.98	0.16	0.87	0.88	0.06	1273.41
8	0.93	0.94	0.93	0.93	0.98	0.98	0.17	0.87	0.87	0.07	1243.27
9	0.93	0.94	0.93	0.93	0.98	0.98	0.17	0.87	0.87	0.07	1095.67
10	0.92	0.93	0.92	0.92	0.97	0.98	0.19	0.85	0.86	0.08	774.83
Average	0.93	0.93	0.93	0.93	0.97	0.98	0.18	0.86	0.86	0.07	1180.71

**Table 7:** Comparison between RAVE model and benchmark models with 95% confidence intervals (*n* = 3000 bootstrap iterations).

Metric	Vanilla RNN (Mean [95% CI])	Naive Bayes (Mean [95% CI])	ResNet (Mean [95% CI])	RAVE (Proposed) (Mean [95% CI])	CI Width (RAVE)
Accuracy	0.865 [0.863, 0.868]	0.767 [0.764, 0.770]	0.901 [0.899, 0.904]	0.928 [0.926, 0.930]	0.003
Precision	0.877 [0.874, 0.880]	0.794 [0.790, 0.797]	0.945 [0.943, 0.947]	0.996 [0.995, 0.996]	0.001
Recall	0.849 [0.845, 0.852]	0.720 [0.715, 0.724]	0.852 [0.849, 0.856]	0.859 [0.856, 0.862]	0.007
F1-Score	0.863 [0.860, 0.865]	0.755 [0.752, 0.758]	0.896 [0.894, 0.898]	0.922 [0.920, 0.924]	0.004
ROC-AUC (Bootstrap)	0.886 [0.884, 0.888]	0.872 [0.869, 0.874]	0.901 [0.899, 0.903]	0.928 [0.926, 0.929]	0.003
PR-AUC	0.865 [0.862, 0.868]	0.858 [0.854, 0.861]	0.879 [0.876, 0.881]	0.926 [0.924, 0.927]	0.004
Log Loss	0.692 [0.692, 0.692]	0.447 [0.443, 0.450]	3.553 [3.479, 3.626]	2.600 [2.539, 2.661]	0.122
Cohen’s Kappa	0.731 [0.726, 0.735]	0.534 [0.528, 0.540]	0.803 [0.799, 0.807]	0.856 [0.852, 0.859]	0.007
MCC	0.731 [0.726, 0.736]	0.537 [0.531, 0.542]	0.807 [0.803, 0.811]	0.864 [0.861, 0.867]	0.006
Hamming Loss	0.135 [0.132, 0.137]	0.233 [0.230, 0.236]	0.099 [0.096, 0.101]	0.072 [0.070, 0.074]	0.003
Brier Score	0.250 [0.250, 0.250]	0.145 [0.144, 0.147]	0.099 [0.096, 0.101]	0.072 [0.070, 0.074]	0.003

**Table 8:** MCID analysis of proposed RAVE model compared with baseline models.

Baseline Model	Metric	RAVE (Proposed) Mean	Baseline Mean	Mean Difference	95% CI	Exceeds MCID
ResNet	Accuracy	0.9323	0.9050	0.0273	[0.0238, 0.0309]	No
ResNet	F1-score	0.9321	0.9040	0.0281	[0.0244, 0.0317]	No
ResNet	ROC-AUC	0.9751	0.9620	0.0131	[0.0098, 0.0164]	No
ResNet	MCC	0.8699	0.8410	0.0289	[0.0246, 0.0331]	No
ResNet	Cohen’s Kappa	0.8650	0.8350	0.0301	[0.0265, 0.0336]	Yes
Vanilla RNN	Accuracy	0.9323	0.9020	0.0304	[0.0268, 0.0340]	Yes
Vanilla RNN	F1-score	0.9321	0.9015	0.0306	[0.0270, 0.0343]	Yes
Vanilla RNN	ROC-AUC	0.9751	0.9605	0.0146	[0.0115, 0.0176]	No
Vanilla RNN	MCC	0.8699	0.8380	0.0318	[0.0276, 0.0360]	Yes
Vanilla RNN	Cohen’s Kappa	0.8645	0.8325	0.0320	[0.0281, 0.0359]	Yes
XGBoost	Accuracy	0.9323	0.8990	0.0334	[0.0296, 0.0370]	Yes
XGBoost	F1-score	0.9321	0.8985	0.0335	[0.0296, 0.0376]	Yes
XGBoost	ROC-AUC	0.9751	0.9580	0.0171	[0.0133, 0.0210]	No
XGBoost	MCC	0.8699	0.8340	0.0359	[0.0316, 0.0403]	Yes
XGBoost	Cohen’s Kappa	0.8645	0.8290	0.0355	[0.0313, 0.0395]	Yes

**Table 9:** Pairwise DeLong ROC-AUC comparisons vs. RAVE (proposed).

Model	ΔAUC (RAVE Model)	z-Value	*p*-Value	Significance
ResNet	+0.0147	4.68	0.000027	Yes
Vanilla RNN	+0.0303	22.39	0.000000	Yes
DBN	+0.0377	59.83	0.000000	Yes
Gradient Boosting	+0.0825	64.77	0.000000	Yes
Logistic Regression	+0.0965	72.93	0.000000	Yes
Naïve Bayes	+0.1068	77.56	0.000000	Yes

**Table 10:** Performance of the proposed RAVE model on the original (unbalanced) test dataset with 95% confidence intervals (bootstrap + DeLong, (n)=3000).

Metric	Mean ± 95% CI
Accuracy	0.8918 ± [0.8906, 0.8930]
Precision	0.8465 ± [0.8428, 0.8501]
Recall	0.8918 ± [0.8906, 0.8930]
F1-Score	0.8566 ± [0.8551, 0.8582]
ROC-AUC (Bootstrap)	0.7760 ± [0.7696, 0.7824]
ROC-AUC (DeLong)	0.7760 ± [0.7680, 0.7841]
PR-AUC	0.2655 ± [0.2564, 0.2746]
Log Loss	0.3049 ± [0.3016, 0.3084]
Cohen’s Kappa	0.0826 ± [0.0722, 0.0933]
MCC	0.1187 ± [0.1043, 0.1332]
Hamming Loss	0.1082 ± [0.1070, 0.1094]
Brier Score	0.0881 ± [0.0871, 0.0890]

**Table 11:** Pairwise DeLong test results for ROC-AUC comparisons among all classifiers. All pairwise differences are statistically significant (p<0.001).

Model 1	Model 2	*p*-Value
Naïve Bayes	Logistic Regression	$4.0\times 10^{-5}$
Naïve Bayes	Gradient Boosting	<$1\times 10^{-5}$
Naïve Bayes	DBN	<$1\times 10^{-5}$
Naïve Bayes	Vanilla RNN	<$1\times 10^{-5}$
Naïve Bayes	ResNet	<$1\times 10^{-5}$
Naïve Bayes	RAVE (Proposed)	<$1\times 10^{-5}$
Logistic Regression	Gradient Boosting	<$1\times 10^{-5}$
Logistic Regression	DBN	<$1\times 10^{-5}$
Logistic Regression	Vanilla RNN	<$1\times 10^{-5}$
Logistic Regression	ResNet	<$1\times 10^{-5}$
Logistic Regression	RAVE (Proposed)	<$1\times 10^{-5}$
Gradient Boosting	DBN	<$1\times 10^{-5}$
Gradient Boosting	Vanilla RNN	<$1\times 10^{-5}$
Gradient Boosting	ResNet	<$1\times 10^{-5}$
Gradient Boosting	RAVE (Proposed)	<$1\times 10^{-5}$
DBN	Vanilla RNN	$1.0\times 10^{-5}$
DBN	ResNet	<$1\times 10^{-5}$
DBN	RAVE (Proposed)	<$1\times 10^{-5}$
Vanilla RNN	ResNet	<$1\times 10^{-5}$
Vanilla RNN	RAVE (Proposed)	<$1\times 10^{-5}$
ResNet	RAVE (Proposed)	<$1\times 10^{-5}$

**Table 12:** Comparison among base models and proposed RAVE model’s performance on CVD dataset.

Model	Accuracy	Precision	Recall	F1-Score	ROC-AUC	PR-AUC	LogLoss	Kappa	MCC	Hammming
ResNet	0.823	0.829	0.827	0.827	0.933	0.946	0.373	0.655	0.657	0.173
Vanilla RNN	0.883	0.883	0.883	0.883	0.956	0.963	0.271	0.765	0.766	0.117
Hybrid (Proposed)	0.924	0.926	0.924	0.924	0.976	0.980	0.187	0.849	0.851	0.075

**Table 13:** Layer-wise configurations of ablation study models.

Model	Layer	Output Shape	Units/Filters	Activation	Dropout	Special Components
MLP	Dense-32	(32,)	32	ReLU	None	Fully connected
	Dense-16	(16,)	16	ReLU	None	Fully connected
	Dense-1	(1,)	1	Sigmoid	None	Output
ResNet	Conv1D-64	(input/2, 64)	64	Tanh	None	SeparableConv1D
	ResBlock-16	(input/2, 16)	16	Tanh	0.5	Skip connections, BatchNorm
	ResBlock-8	(input/4, 8)	8	Tanh	0.5	Skip connections, BatchNorm
	GAP	(8,)	N/A	N/A	None	Global Average Pooling
	Dense-1	(1,)	1	Sigmoid	None	Output
VRNN	Reshape	(1, input)	N/A	N/A	None	Pseudo-sequence input
	SimpleRNN-32	(1, 32)	32	Tanh	0.2	Recurrent layer
	SimpleRNN-16	(16,)	16	Tanh	0.2	Recurrent layer
	Dense-1	(1,)	1	Sigmoid	None	Output
RAVE (Proposed)	Conv1D-64	(input, 64)	64	Tanh	None	SeparableConv1D
	ResBlock-32	(input, 32)	32	Tanh	0.2	Skip connections, BatchNorm
	ResBlock-16	(input/2, 16)	16	Tanh	0.2	Skip connections, BatchNorm
	SimpleRNN-32	(input/2, 32)	32	Tanh	0.2	Sequential modeling
	SimpleRNN-16	(16,)	16	Tanh	0.2	Sequential modeling
	Dense-1	(1,)	1	Sigmoid	None	Output

**Table 14:** Hyperparameters used in ablation study models.

Hyperparameter	MLP	ResNet	Vanilla RNN	Proposed RAVE (ResNet+RNN)
Optimizer	Adam	Adam	Adam	Adam
Learning Rate	0.001	0.001	0.001	0.001
Loss Function	Binary Crossentropy	Binary Crossentropy	Binary Crossentropy	Binary Crossentropy
Batch Size	64	64	64	64
Epochs	10	10	10	10
Weight Initialization	Glorot Uniform	He Normal	Glorot Uniform	He Normal
Gradient Clipping	None	None	None	1.0
Input Shape	(input_dim,)	(input_dim,1)	(input_dim,)	(input_dim,1)
Regularization	None	None	None	L2 = $1\times 10^{-4}$ (optional)
Dropout Rate	None	0.5	0.2	0.2

**Table 15:** Performance comparison of models in ablation study.

Metric	MLP (SGD)	MLP (Adam)	ResNet	Vanilla RNN	Proposed RAVE
Accuracy	0.839	0.9106	0.891	0.850	0.921
Precision	0.840	0.9113	0.901	0.850	0.926
Recall	0.839	0.9106	0.891	0.849	0.921
F1-score	0.839	0.9106	0.890	0.849	0.920
ROC-AUC	0.916	0.9699	0.961	0.938	0.971
PR-AUC	0.910	0.9752	0.968	0.946	0.977
Log Loss	0.371	0.2133	0.254	0.311	0.192
Cohen’s Kappa	0.679	0.8212	0.781	0.699	0.841
MCC	0.679	0.8219	0.792	0.700	0.847
Hamming Loss	0.161	0.0894	0.110	0.151	0.079
Execution Time (s)	283.13	314.05	368.10	195.38	498.71

## Data Availability

A publicly available dataset was analyzed in this study. The dataset can be found at: https://www.kaggle.com/datasets/alexteboul/heart-disease-health-indicators-dataset (accessed on 5 November 2025).

## Abbreviations

The following abbreviations are used in this manuscript:

AI	Artificial Intelligence
BRFSS	Behavioral Risk Factor Surveillance System
CNN	Convolutional Neural Network
DL	Deep Learning
DBN	Deep Belief Network
FCV	Fold Cross-Validation
GB	Gradient Boosting
HD	Heart Disease
HDNN	Hybrid Deep Neural Network
LE	Label Encoder
LoRAS	Localized Random Affine Shadow Sampling
LR	Logistic Regression
MCC	Matthews Correlation Coefficient
ML	Machine Learning
NB	Naive Bayes
PR-AUC	Precision–Recall Area Under the Curve
ProWRAS	Proximity Weighted Random Affine Shadow Sampling
RFE	Recursive Features Elimination
ResNet	Residual Network
SHAP	SHapley Additive exPlanations
Vanilla RNN	Vanilla Recurrent Neural Network
XAI	EXplainable Artificial Intelligence
